# Enrichment and Proteomic Profiling of Stromal‐Associated Extracellular Vesicle Subpopulations From Arthritic Synovial Fluid by Size‐Exclusion Chromatography Coupled to CD90‐Directed Immunocapture

**DOI:** 10.1002/jev2.70353

**Published:** 2026-09-30

**Authors:** Stefanie Kurth, Edveena Hanser, Simone Häner‐Massimi, Stavros Giaglis, Florian Geier, Dominik Burri, Katarzyna Buczak, Ute Heider, Stefan Wild, Yves P. Acklin, Christian Egloff, André N. Tiaden, Diego Kyburz

**Affiliations:** ^1^ Experimental Rheumatology University of Basel Basel Switzerland; ^2^ Department of Biomedicine University of Basel Basel Switzerland; ^3^ Bioinformatic Core Facility University of Basel Basel Switzerland; ^4^ SIB Swiss Institute of Bioinformatics Basel Switzerland; ^5^ Proteomic Core Facility Biozentrum University of Basel Basel Switzerland; ^6^ Miltenyi Biotec Bergisch Gladbach Germany; ^7^ University of Basel Basel Switzerland; ^8^ Department of Surgery Kantonsspital Graubünden Chur Switzerland; ^9^ Department Knee Surgery Schulthess Klinik Zürich Switzerland; ^10^ Department of Rheumatology University Hospital Basel University of Basel Basel Switzerland

**Keywords:** arthritis, biomarker, EV separation, EV subpopulation, proteomics, synovial fluid

## Abstract

Extracellular vesicles (EVs) are emerging as key mediators of disease‐associated intercellular communication and as promising biomarker sources across many disease conditions, including joint disorders such as rheumatoid arthritis (RA) and osteoarthritis (OA). Molecular profiling of synovial tissue has uncovered disease‐driving cellular states, but tissue biopsies are invasive and not routinely available. EVs in synovial fluid may offer a minimally invasive and complementary window into joint pathobiology. However, the molecular complexity of synovial fluid and the heterogeneity of EV populations have hindered the analysis of defined disease‐relevant EV subsets. To address this challenge, we developed a refined size‐exclusion chromatography‐ultrafiltration (SEC‐UF) workflow coupled to magnetic bead‐based immunocapture for targeted enrichment of cell type‐associated EV subpopulations from arthritic synovial fluid. As proof‐of‐concept, we targeted a stromal‐associated EV population using CD90/THY1, a surface marker expressed by discrete synovial fibroblasts subsets implicated in arthritis pathobiology. In vitro validation using synovial fibroblast‐derived EVs confirmed surface‐accessible CD90 and demonstrated selective immunocapture of defined EV subpopulations. Application to patient‐derived synovial fluid showed that SEC‐UF pre‐enrichment improves the robustness of CD90^+^ EV recovery from this complex biofluid. Liquid chromatography‐tandem mass spectrometry (LC‐MS/MS) profiling established broad EV‐associated proteome coverage in synovial fluid (SF)‐EV preparations and identified multiple proteins reflecting the inflamed arthritic synovial environment. Fraction‐resolved proteomics enabled comparison of the CD90‐immunocaptured SF‐EV subset with the non‐captured CD90^−^/CD90^low^ SF‐EV pool. Differential proteomics and surfaceome‐informed cell‐of‐origin analysis supported enrichment of stromal‐associated surface markers in the CD90^+^ fraction, whereas the non‐captured SF‐EV pool retained broader immune‐associated and residual stromal EV inputs. Together, this fit‐for‐purpose workflow provides a strategy to resolve EV heterogeneity patient synovial fluid and supports future biomarker‐oriented studies of defined subpopulations in arthritic diseases.

## Graphical abstract

Synovial fluid contains heterogeneous EV populations reflecting stromal and immune‐associated cellular inputs from the arthritic joint. SEC‐UF enrichment coupled to CD90‐targeted immunocapture enables recovery of stromal‐associated EV subpopulation from complex patient synovial fluid, while focused LC‐MS/MS and cell‐of‐origin analysis enable molecular characterization, proteome coverage assessment, and biological contextualization of fractionated EVs. This refined workflow provides a methodological and analytical approach for resolving EV heterogeneity in synovial fluid and supports future liquid biopsy‐oriented biomarker studies of defined EV subpopulations in arthritis.

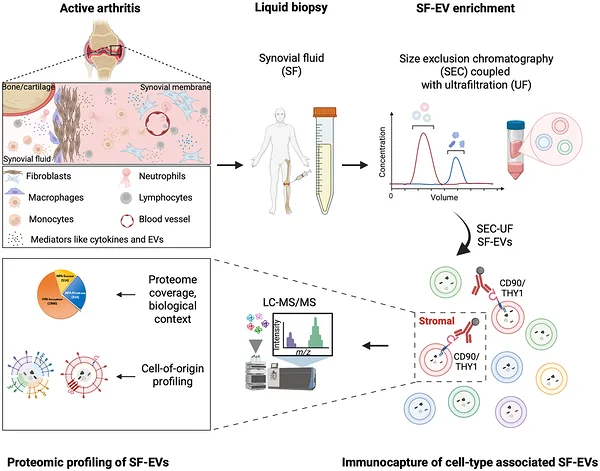

## 1 Introduction

Extracellular vesicles (EVs) represent a heterogenous ensemble of nanosised membrane‐bound particles ubiquitously released by diverse cell types (van Niel et al. 2018). Research in the last decade established EVs as instrumental entities in intercellular communication which facilitate component exchange between cells in their vicinity but also convey functional cargo to distant tissues and organs (van Niel et al. 2022; Kalluri and LeBleu 2020). EVs are pivotal in mediating a broad spectrum of biological processes including differentiation, proliferation and regeneration, or supporting cellular and tissue homeostasis by quickly adapting to environmental changes (Yanez‐Mo et al. 2015). Recently, immunomodulatory functions were attributed to EVs derived from the immune system, including antigen presentation, activation of immune cells, regulation of inflammation and immune surveillance, thereby underscoring their significance in health and disease (Aloi et al. 2024; Veerman et al. 2019; Buzas 2023; Yates et al. 2022). As such, EVs are implicated in the aetiology of numerous pathological conditions involving the immune system, particularly in cancer and in complex diseases originating from dysfunctional immune regulation (Marar et al. 2021; Kalluri and McAndrews 2023). In chronic joint diseases such as rheumatoid arthritis (RA) and osteoarthritis (OA), EVs have been linked to disease‐associated intercellular communication and can contribute to processes involved in inflammation, tissue remodelling and disease progression (Schioppo et al. 2021; Miao et al. 2022; Fan et al. 2022). Consequentially, EVs are increasingly explored as therapeutic targets and promising biomarker candidates, offering novel perspectives for identifying disease‐relevant mechanisms, monitoring disease activity or assessing therapeutic responses (Cheng and Hill 2022; Hoshino et al. 2020; Asleh et al. 2023). Their biomarker potential is closely related to the unique molecular composition of membrane‐bound proteins and intravesicular biomolecules, which both reflect the cellular identity and the physiological or pathological state of cells and tissues from which they originate (Hallal et al. 2022). EVs are anticipated to carry molecular signatures that provide a unique perspective on the cellular dynamics in the inflamed tissue (Huang et al. 2022). In arthritis, analysing the functional surfaceome and encapsulated cargo of EVs released into synovial fluid may offer a minimally invasive window into the cellular and molecular processes occurring within the inflamed joint, thereby providing a real‐time snapshot of disease activity and progression, with significant implications for diagnosis, prognosis and monitoring of therapeutic responses.

Recent advances in molecular profiling of synovial tissue by multimodal single‐cell omics approaches (Pandey and Bhutani 2024), have revealed distinct stromal, myeloid and lymphoid cell states associated with histopathological endotypes linked to disease activity and treatment outcomes in arthritis (Weisenfeld et al. 2024; Rivellese et al. 2023; Lakhanpal et al. 2021; Lewis 2024; Bykerk 2023). These studies have substantially advanced the understanding of the cellular heterogeneity of the arthritic synovium. However, a direct clinical translation of tissue‐resolved synovial endotypes as disease indicators remains limited by the need for invasive surgical intervention or needle biopsies to obtain synovial tissue from inflamed joints (Pitzalis et al. 2013; Humby et al. 2015; Orr et al. 2017; Veale 2019). Liquid biopsies, such as synovial fluid from the joint cavity, offer a less invasive alternative for capturing disease‐associated molecular information for biomedical research or clinical use (Bo et al. 2023; Mahendran et al. 2017). Synovial fluid is in direct contact with the inflamed synovial membrane and contains EVs actively released by tissue‐resident cells as well as infiltrating immune cells located within the periarticular tissue (Schioppo et al. 2021; Zhang, Duan, et al. 2023; Boere et al. 2018). Thus, synovial fluid (SF)‐EVs may provide molecular information complementary to synovial tissue or direct synovial fluid profiling, particularly if EV subpopulations can be linked to defined cellular sources.

However, the application of SF‐EVs for biomarker discovery in arthritis is technically challenging. Synovial fluid is a complex, protein‐rich biofluid with lubricant properties, abundant extracellular matrix (ECM) components, serum‐derived proteins and variable viscosity, all of which can affect EV recovery, purity and downstream analysis (Ben‐Trad et al. 2022; Urzi et al. 2022; Foers et al. 2018; Lehrich et al. 2021). In addition, EVs derived from patient synovial fluid represent a heterogenous mixture of different vesicle populations that vary in size, density, biogenesis, lipid composition, surface marker expression and molecular content of encapsulated cargo (Vagner et al. 2019). Bulk EV isolation approaches therefore risk to mask disease‐relevant but less abundant EV subsets and complicate attribution of molecular signatures to specific cell types or tissue compartments. Moreover, co‐isolation of soluble proteins, protein aggregates, ECM fragments and EV‐associated corona components can interfere with quantitative and qualitative interpretation of SF‐EV preparations, particularly in mass spectrometry (MS)‐based proteomics.

To address these challenges, we developed a fit‐for‐purpose EV enrichment strategy that combines size exclusion chromatography and ultrafiltration (SEC‐UF) with nanosised magnetic bead‐based immunocapture. This approach was designed to reduce the complexity of synovial fluid‐derived EV preparations while enabling targeted enrichment of cell type‐associated EV subpopulations. As a proof‐of‐concept, we focused on CD90/THY1, a stromal‐associated surface marker expressed by synovial fibroblast subsets implicated in arthritis pathobiology. Using Western blotting, LC‐MS/MS proteomics and surfaceome‐informed cell‐of‐origin analysis, we assessed whether CD90‐targeted immunocapture enriches stromal‐associated EVs and separates them from a broader non‐captured SF‐EV pool. This workflow provides an analytical framework for resolving EV heterogeneity in patient synovial fluid and supports future biomarker‐oriented studies of defined EV subpopulations in arthritic diseases.

## 2 Methods

### 2.1 Patients and Collection of Bio‐Samples

Synovial fluid samples were obtained from patients with different forms of inflammatory and degenerative arthritis, including RA, OA or crystal arthropathies, treated at the Department of Rheumatology, University Hospital Basel, Switzerland. Collected synovial fluid was centrifuged at 2000 × *g* for 10 min at RT to remove cells and debris, aliquoted and stored at –80°C until use. Synovial tissues were provided by the Department of Orthopedics and Department of Rheumatology, University Hospital Basel. Synovial tissue biopsies from surgical interventions were temporally stored in Tissue Storage Buffer at 4°C (Miltenyi) for subsequent dissection, homogenisation and isolation of a single cell suspension for further experiments. Patient characteristics are listed in Table S1. All patients gave informed consent, and the study was approved by the local ethics review board (EKNZ 2019‐01693).

### 2.2 Isolation of Cells, In Vitro Cell Culture and Macrophage Differentiation

Synovial fibroblasts were isolated from RA‐ and OA‐patient synovial tissue as previously described (Kyburz et al. 2003). Briefly, synovial tissue biopsies stored in Tissue Storage Buffer were washed with PBS and synovial membrane parts were manually dissected into small tissue pieces (2–5 mm^2^). Collected pieces were washed with PBS and submitted to automatically dissociation using the gentleMACS Octo device with the Human Tumor Dissociation kit (both Miltenyi) to generate a single cell suspension. Isolated cells were then transferred to in vitro cell culture to select for and expand synovial fibroblasts. For macrophages, the THP1 monocyte cell line (Merck/Sigma–Aldrich) or monocyte‐derived M1 macrophages from peripheral blood of healthy donors (Blood Donation Center, University Hospital Basel) were used. All cells were maintained in an incubator at 37°C, 5% CO_2_ and cultured in RPMI‐1640 medium supplemented with 10% FCS (Gibco/ThermoFisher Scientific), 1% Penicillin/Streptomycin and 25 mM HEPES. Synovial fibroblasts were passaged all 10–14 days depending on cell density, while THP1 monocytes were passaged when cells reached a density of 1 × 10^6^ cells/mL. For all experiments, cell types were used during passages 2–10.

For differentiating THP1 monocytes to macrophages, 6 × 10^4^ THP‐1 cells were plated in 96 Well plate using complete RPMI‐1640 with 10 ng/mL of phorbol myristate acetate (PMA). After 24 h PMA stimulation, attached cells were washed with fresh medium and used for further experiments. The differentiation of CD14/CD16 monocytes isolated from peripheral blood mononuclear cells (PBMC) of healthy donors toward an M1‐like macrophage type was performed by culturing cells in complete medium supplemented with recombinant human GM‐CSF (50 ng/mL, Peprotech/ThermoFisher Scientific) for up to 7 days, as previously published (Quero et al. 2019).

### 2.3 Preparation of Cell Culture Conditioned Medium (CM) and Synovial Fluid for EV Isolation

Cell culture‐derived conditioned medium (CM) and patient synovial fluid display different sample matrix‐complexity, which require distinct preparatory steps for efficient downstream EV isolation. The two sample specimens differ also substantially in collection volume, protein content and viscosity. Cell culture‐derived CM was harvested in large volume of approximately 100–200 mL to yield decent EV concentrations, and therefore required a pre‐concentration step using Amicon Ultra centrifugal units (15×, 100 kDa cut off, Millipore) before EV isolation. In contrast, patient synovial fluid was available only in limited volumes but contains high EV concentrations, abundant proteins and matrix components, which required enzymatic pre‐treatment and dilution to generate an appropriate input sample for efficient EV isolation.

Cell culture‐derived EVs were isolated from CM of in vitro cultured synovial fibroblasts of arthritis patients or from differentiated THP1 macrophages. For isolation of synovial fibroblast‐derived EVs, 4 × 10^6^ cells were seeded per T175 flask and incubated for 72 h (37°C, 5% CO_2_). Afterward, cells were washed with PBS and the medium replaced with 30 mL exosome‐depleted FBS medium (Gibco/ThermoFisher Scientific). The CM was collected after 48 h incubation. For the isolation of THP1 macrophage‐derived EVs, 30 × 10^6^ THP1 monocytes were seeded per T175 flask and differentiated into macrophages with PMA (10 ng/mL) for 24 h (37°C, 5% CO_2_). Then, the differentiated THP1 macrophages were washed three times with PBS and stimulated with 30 mL exosome‐depleted FBS medium supplemented with LPS (100 ng/mL, InvivoGen) and INFγ (20 ng/mL, Peprotech/ThermoFisher Scientific). The CM was collected after 24 h incubation. Before the SEC‐UF‐based EV isolation, CM collected from 3 to 6× T175 flasks (30 mL/T175) were pooled and processed by sequential centrifugation at 500 × *g* for 10 min and 3000 × *g* for 10 min at RT to remove dead cells and cellular debris. Then, the pooled CM was concentrated via UF using Amicon Ultra centrifugal units (15×, 100 kDa cut off, Millipore) to yield an appropriate input sample volume of 2 mL for subsequent SEC processing.

Synovial fluid samples underwent a multistep pre‐treatment procedure to reduce viscosity, minimise interference caused by abundant ECM components, and to eliminate cellular debris and extracellular DNA/protein aggregates, as recommended by the ISEV Synovial Fluid Task Force and in line with the MISEV 2023 guidelines (Welsh et al. 2024). An aliquot of pre‐cleared synovial fluid stored at –80°C was thawed on ice and treated with hyaluronidase (30 U/mL; Type VI‐S, Merck/Sigma–Aldrich) and DNAse I (20 U/mL, Roche), and incubated for 45 min at 37°C with gentle agitation. A protease/phosphatase inhibitor cocktail (1:100 v/v; Halt protease inhibitor cocktail, Thermo Fisher Scientific) was added during the incubation step to preserve protein integrity. After enzymatic digestion, the synovial fluid was centrifuged at 1000 × *g* for 5 min at RT and cleared supernatant was used for subsequent individual EV isolation protocols.

### 2.4 Isolation of Synovial Fluid EVs by Ultracentrifugation (UC)

UC‐based isolation was used for exploratory bulk SF‐EV pre‐screening. A 1 mL synovial fluid aliquot was pre‐treated as mentioned above and diluted 1:5 in filtered PBS (0.22 µm). EVs were pelleted via UC at 100,000 x *g* for 1 h at 4°C using an Optima XPN‐90 ultracentrifuge (Beckman Coulter). Resulting pellets were carefully resuspended in 50 µL RIPA‐Buffer supplemented with protease/phosphatase inhibitors (1:100 v/v) for subsequent protein analysis.

### 2.5 Isolation of Synovial Fluid EVs by Size Exclusion Chromatography and Ultrafiltration (SEC‐UF)

A 2 mL pre‐treated synovial fluid sample was first filtered using a filter syringe (0.45 µm) and subsequently diluted 1:2 in filtered PBS (0.22 µm) before subjected to EV isolation via SEC using a qEV1 Gen 2 35 nm column (IZON) according to the manufacture's protocol. During SEC, the EV‐rich fractions 5–9 were collected and pooled followed by UF using Amicon Ultra centrifugal units (14×, 10 KDa cut off, Millipore) to concentrate the isolated EVs to a final volume of 1 mL.

### 2.6 Isolation of Cell Culture Conditioned Medium (CM) EVs by SEC‐UF

The SEC‐UF‐enrichment of A 2 mL pre‐processed CM sample was subjected to EV isolation via SEC using a qEV1 Gen2 35 nm column (IZON) according to the manufacture's protocol. During SEC, the EV‐rich fractions 5–9 were collected and pooled followed by UF using Amicon Ultra centrifugal units (14×, 10 KDa cut off, Millipore) to concentrate the isolated EVs to a final volume of 1 mL.

### 2.7 EV Characterisation

The particle concentration (particle/mL) of all SEC‐UF‐isolated EVs was measured using the nanotracking analyzer (NTA) ZetaView PMX 110 V3 (ParticleMetrix). EV samples were diluted in filtered PBS and injected into the laser chamber, assessing the particle concentration measured by analysing the Brownian motion of each particle. NTA‐software ZetaView (version 8.05.14 SP7) was used to evaluate of the data and determine the particle concentration.

Nano flow cytometry using the NanoFlow Analyzer nFCM (NanoFCM Inc.) was applied to assess single‐vesicle surface staining and antibody accessibility of selected immunocapture target markers. nFCM‐derived particle counts and size distributions were additionally used as an orthogonal validation of NTA‐based concentration/size estimates, particularly because NTA measurements can be influenced by co‐isolated non‐EV particles in complex biofluid‐derived preparations. Particle size and concentration were calibrated using a standardised nanoparticle mixture of defined sizes (40–200 nm beads, silica nanosphere cocktail, S16M‐Exo). For surface staining, 9 µL EVs (2 × 10^10^ EVs/mL) were incubated with 1 µL PE‐conjugated antibody, including anti‐CD63_PE (BD, 556020), anti‐CD90_PE (Miltenyi, 130‐114‐903), anti‐Podoplanin_PE (Miltenyi, 130‐117‐687) or Isotype_PE (Control, Miltenyi, 130‐113‐438) for 30 min at RT in the dark under native conditions without fixation or permeabilisation. To reduce background noise and remove potential antibody‐aggregates, all antibodies were centrifuged at 17,000 × *g* for 10 min at 4°C. For nFCM, the 10 µL staining mixtures were further diluted with filtered PBS (1:100 for CD63 staining, or 1:25 for CD90, PDPN staining) to allow optimal signal acquisition by aiming for 2000–12,000 events/min. A set of staining control samples were used according to the MISEV guidelines (Welsh et al. 2024), including filtered PBS_only, antibody_only, unstained EVs, isotype antibody staining and NP40 control. All samples were equally treated. NP40 control was performed by adding 40 µL of 2% NP40 in PBS after the staining and incubating for 30 min at RT in the dark. nFCM software (NF profession 1.0) was used to acquire and analyze data. Fluorescence signal gates were set based on auto‐thresholding the baseline signal of the matched PBS controls.

Morphology and quality of isolated EVs was assessed by transmission electron microscopy (TEM). Five microlitres of EV suspension (1 × 10^9^ EVs/mL SEC‐UF‐isolated EVs; or neat concentration of eluate and flowthrough fraction; concentration unknown of immunocaptured fractions) was adsorbed onto glow‐discharged, carbon‐coated copper grids (400‐mesh copper grids with Parlodion carrier film, 50 nm thick and vapour‐deposited carbon, 10 nm thick) and incubated for 60 s. For negative staining, the grids were rinsed briefly with ddH_2_O (50 µL each) and stained with two drops of 2% uranyl acetate aqueous solution (staining duration was 10 s). Grids were blotted with filter paper, air‐dried and examined using a CM100 Phillips TEM, operating at 80 kV.

### 2.8 EV Precipitation, Protein Lysis and Measurement of Protein Concentration

To prepare EV protein for assessing protein concentration and Western blot analysis we precipitated the collected EV preparation using the Exosome Isolation Kit (Total Exosome Isolation Reagent from cell culture media, ThermoFisher Scientific) according to the manufacturer's protocol. In brief, a defined volume of EV suspension was mixed with the precipitation solution at the ratio of 1:1, vortexed and incubated overnight at 4°C. Then, the sample was centrifuged at 10,000 × *g* for 1 h at 4°C, the resulting pellet was washed with PBS and lysed in RIPA‐Buffer (Pierce/Thermo Fisher Scientific) supplemented with phosphatase/protease inhibitors (1:100 v/v). The protein concentration was measured using the Micro‐BCA‐Protein Assay Kit (Thermo Fisher Scientific) according to the manufacturer's protocol.

### 2.9 Membrane Protein Extraction From SEC‐UF EV Preparations

To confirm the surface localisation of CD90 and PDPN on EVs, membrane and cytosolic protein fractions were separated using the Mem‐PER Plus Membrane Protein Extraction Kit according to the manufacturer's protocol (Thermo Fisher Scientific), with slight adaptation for EV material. Briefly, 2 × 10^10^ SEC‐UF‐isolated EVs were precipitated as described before. The resulting pellet was resuspended in 200 µL proprietary permeabilisation buffer supplemented with protease/phosphatase inhibitors (1:100 v/v), incubated for 10 min at 4°C with constant shaking followed by centrifugation at 16,000 × *g* for 15 min at 4°C to separate the cytosolic fraction (supernatant) and the membrane fraction (pellet). The supernatant was transferred into a new tube, while the pellets were resuspended in 100 µL solubilisation buffer supplemented with protease/phosphatase inhibitors (1:100 v/v) and incubated for 30 min at 4°C with constant shaking. Following centrifugation at 16,000 × *g* for 15 min at 4°C, the supernatant including the solubilised membrane and membrane‐associated fraction was collected into a new tube. The same protocol was also used to extract membrane proteins from cultured synovial fibroblasts. In brief, 2.5 × 10^6^ cultured synovial fibroblasts were harvested by scraping the cells in cold PBS, followed by centrifugation at 300 × *g* for 10 min at 4°C to pellet the cells. The cell pellet was lysed in 375 µL proprietary permeabilisation buffer supplemented with protease/phosphatase inhibitors (1:100 v/v) and processed as described above, with the exception of adding 250 µL of solubilisation buffer to the pelleted membrane fraction.

### 2.10 EV Protein Detection by Western Blot

Western blotting was performed to evaluate marker enrichment and bead separation specificity. SEC‐UF‐isolated bulk EVs and bead‐separated EV fractions were precipitated as described before. The resulting pellet was resuspended and lysed in RIPA‐buffer containing phosphatase/protease inhibitors (1:100 v/v), and protein concentration was assessed as described above. Subsequently, samples were denatured by adding DTT (50 mM final concentration) in Lämmli buffer and heated for 5 min at 70°C. Samples were cooled on ice and loaded on a 4%–15% SDS gradient gel (Mini‐PROTEAN TGX Gel, BioRad) for separating the proteins according to their molecular weight. If not stated otherwise, for samples derived from the immunocapture experiments, including the eluate (E), wash (W) and flowthrough (FT), the complete protein amount was loaded. In other experiments, equal amounts of proteins were loaded as indicated in the corresponding figure legends. The proteins were blotted on PVDF membrane using the Trans‐Blot Turbo system (BioRad). The membrane was blocked in blocking buffer (5% milk in TBS with 0.05% Tween20 [TBS‐T]) for 1 h at RT, followed by three washing steps for 5 min in washing buffer (TBS‐T) and an overnight incubation with primary antibodies at 4°C (CD90 (CST‐13801S‐Rabbit; 1:1000), PDPN (CST‐9047‐Rabbit; 1:1000), CD68 (UltraMAB‐UM870047‐Mouse; 1:2000), CD63 (Invitrogen‐10628D‐Mouse; 1:1000), CD9 (Invitrogen‐10626D‐Mouse; 1:1000), CD81 (Invitrogen‐10630D‐Mouse; 1:1000), CD163 (CST‐93498‐Rabbit; 1:1000), Syntenin‐1 (Abcam‐ab133267‐Rabbit; 1:1000), Flotillin‐1 (BD‐610820‐Mouse; 1:1000), Annexin‐1 (BD‐610067‐Mouse; 1:5000), Calnexin (CST‐2679S‐Rabbit; 1:1000), CD48 (CST‐29499T‐Rabbit; 1:1000)). After three washing steps, HRP‐coupled secondary antibodies were solved in blocking buffer and incubated for 1 h at RT with constant shaking (Goat‐anti‐Rabbit [CST‐6211234; 1:3000], Horse‐anti Mouse [CST‐7076S; 1:3000]). Antibody‐stained membranes were washed three times and finally rinsed with TBS. The chemiluminescence signals were visualised using Pierce ECL Western Blotting Substrate (Thermo Fisher Scientific) and recorded with the Fusion FX imager (Vilber).

Western Blot quantifications were performed on *n* ≥ 3 biological replicates (independent samples). Band signal intensities (integrated pixel density) of the protein of interest were quantified using Fiji/ImageJ (Version 2.14.0/1.54f) with two technical replicate measurements per signal. In each lane, the band signal intensity was normalised to the corresponding syntenin‐1 band to enable relative EV marker comparisons within the same experiment (i.e., to account for variation in EV‐associated protein content between fractions/samples). Syntenin‐1 was used as an EV‐associated reference protein and not to infer equal EV particle numbers across donors. Where applicable, membranes were re‐probed and normalisation was performed using bands from the same membrane and exposure conditions. Cell lysates from cultured synovial fibroblasts and differentiated M1‐like macrophages (derived from healthy donor PBMCs) were included as positive controls for antibody reactivity and marker identity, and to confirm expected cell‐type‐associated expression.

### 2.11 Marker‐Directed Immunocapture of EVs Using Prototype Nanosised Magnetic Beads

Magnetic immunocapture experiments were performed using prototype 50 nm anti‐CD90 and anti‐PDPN magnetic beads (kindly provided by Miltenyi Biotec) and a modified µMACS separation protocol. Isotype control beads and a commercial EV isolation kit targeting CD63, CD9 and CD81, (human EV Isolation KIT Pan, both Miltenyi) were included as controls. In brief, 50 µL of the bead‐coupled antibodies were added to EV preparations isolated from different source material (see below, individual immunocapture setups). The EV/antibody mixture was incubated for 1 h at RT with mild agitation. Subsequently, the bead‐EV complexes or control samples were loaded onto µMACS separation columns allowing to collect the flowthrough (FT; non‐bound EVs), the wash (W; unspecific proteins) and two consecutive eluates 1 and 2 (E1 and E2) harbouring the targeted EV population. E2 was yielded by an additional elution step applying 100 µL fresh isolation buffer to check the efficiency of the first elution and approximate the remaining material on the column.

Dependent on the individual immunocapture setup, the current protocol included some modifications: For approaches using synovial fluid as source material (spike‐in experiments, immunocapture using synovial fluid‐derived SEC‐UF EVs or direct immunocapture using pre‐treated synovial fluid) the FT was subjected a second time to a fresh magnetic column and the resulting additional eluate was pooled to the existing eluates from the first separation run. This additional step reduced the number of remaining beads in the FT fraction and also increase the portion of marker‐positive EVs in the final eluate collection. In the experiments using pre‐treated synovial fluid directly as immunocapture input, the collected FT and W fractions were subjected to an additional SEC‐UF step, with the aim to minimise the dominant portion of non‐EV proteins in these fractions. This step was necessary to visualise the proportion of EV‐associated protein in the FT and W fraction otherwise obscured by the high abundant serum protein content in the synovial fluid samples without prior SEC‐UF EV enrichment. Finally, all fractions, the FT, W and pooled E, were precipitated as described above, and magnetic beads were removed by sequential centrifugation steps (2 × 30 min, 1 × 10 min, both at 10,000 × *g* at 4°C).

### 2.12 Immunocapture‐Based Fractionation of SEC‐UF EVs Derived From Cell Culture CM

A sample of 2 × 10^10^ SEC‐UF‐isolated EVs were used as input for the immunocapture approach using anti‐CD90, anti‐PDPN and Isotype control magnetic beads. For the mixed population experiments, 2 × 10^10^ SEC‐UF‐isolated EVs derived from cultured synovial fibroblasts (CD90^+^) or THP1‐macrophages (CD68^+^) were mixed at a ratio of 1:1 yielding a final concentration of 4 × 10^10^ EVs as input source. The immunocapture of CD90^+^ EVs from the mixed EV suspension using a modified µMACS separation protocol was performed as described above. Yielded E, W and FT fractions were precipitated as described before. For Western blot analysis, resulting pellets were lysed in 20–30 µL RIPA‐Buffer supplemented with phosphatase/protease inhibitors (1:100 v/v).

### 2.13 Spiking Human Synovial Fluid With SEC‐UF EVs Derived From Cultured Synovial Fibroblasts

2 × 10^10^ SEC‐UF EVs in 500 µL filtered PBS isolated from cultured synovial fibroblast CM were spiked into an aliquot of 500 µL pre‐treated synovial fluid using a patient sample lacking endogenous CD90^+^ EVs (based on Western blot assessment). The spiked synovial fluid sample was diluted 1:1 with filtered PBS to a final volume of 2 mL with the aim to reduce sample viscosity and interference effects during the immunocapture procedure due to non‐EV proteins. The 2 mL were applied as input for the subsequent immunocapture using prototype anti‐CD90 and Isotype control beads as describe above. The pellets following precipitation of FT, W and E fractions were lysed in 20–30 µL RIPA‐buffer supplemented with protease/phosphatase inhibitors (1:100 v/v).

### 2.14 Immunocapture‐Based Fractionation of Synovial Fluid‐Derived EVs

Patient‐derived synovial fluid was pre‐treated as described before, then 2 mL of pre‐treated synovial fluid was either subjected to EV isolation/enrichment via SEC‐UF and subsequent immunocapture, or used as input material for the direct immunocapture approach. The SEC‐UF isolation of 2 mL pre‐treated synovial fluid yielded 1 mL EV suspension in PBS with differing concentrations ranging between 1 × 10^10^ and 4 × 10^11^ particles/mL dependent on the individual patient sample. The complete 1 mL EV sample was subjected to the immunocapture separation procedure as described above. Yielded E, W and FT fractions were precipitated as described before. The resulting pellets were lysed in 30–40 µL RIPA‐buffer supplemented with phosphatase/protease inhibitors (1:100 v/v) for subsequent Western blot analysis.

For the direct immunocapture approach, the 2 mL pre‐treated synovial fluid sample was diluted 1:4 in filtered PBS to minimise adverse effects for µMACS column performance due to synovial fluid viscosity. The diluted sample was processed in four individual immunocapture runs (2 mL input each) and the yielded fractions precipitated as described above. Finally, all four individual pellets from each fraction were pooled and lysed in 40 µL RIPA‐buffer supplemented with protease/phosphatase inhibitors (1:100 v/v).

### 2.15 Mass Spectrometry (MS)‐Based Proteomic Measurement, Analysis and Data Processing

LC‐MS/MS analysis was applied to assess EV proteomics. To prepare EV isolates with adequate quality and purity for quantitative proteomics, SEC‐UF‐enriched EVs coupled to the established immunocapture approach was used as described in the previous paragraphs with some modifications. The EV‐bead complexed samples were loaded onto µMACS columns, and flowthrough (FT) collection and washing steps were performed according to manufacturer's guidelines. Then, then the retained bead‐bound EVs were directly lysed on column by temporarily locking the column at the separation magnet and incubating the retained EVs with 100 µL lysis buffer (5% SDS, 50 mM Tris, pH 7.8) for 40 min at RT. Then, the column was released from the magnet and the lysed material eluted by adding additional 100 µL lysis buffer. This eluate (E) and the corresponding FT fraction were precipitated as described before and the resulting pellet resuspended in 200 µL lysis buffer. To remove residual beads from the precipitated E and FT fraction, the samples were centrifuged 2× for 40 min at 10,000 × *g* at RT and the resulting darkish bead‐pellet removed. Then, the E and FT fraction were boiled for 5 min at 95°C and stored at –20°C until further preparation for proteomic analysis.

Thawed E and FT fractions were adjusted with SDS and TEAB (Triethylammonium bicarbonate) to the final concentration of 5% and 100 mM, respectively. Samples were reduced by addition of TCEP (Tris‐2(carboxyethyl)phosphine) to the final concentration of 10 mM and 10 min incubation at 95°C. Proteins were then alkylated with 20 mM iodoacetamide for 30 min at RT (protected from the light). Then, samples were digested using S‐Trap micro spin columns (Protifi) according to the manufacturer's instructions. Shortly, 12% phosphoric acid was added to each sample (final concentration of phosphoric acid 1.2%) followed by the addition of S‐trap buffer (90% methanol, 100 mM TEAB pH 7.1) at a ratio of 6:1. Samples were mixed by vortexing and loaded onto S‐trap columns by centrifugation at 4000 × *g* for 1 min followed by three washes with S‐trap buffer. Digestion buffer (50 mM TEAB pH 8.0) containing sequencing‐grade modified trypsin (Promega) was added to the S‐trap column and samples were incubated for 1 h at 47°C. Peptides were eluted by the consecutive addition and collection by centrifugation at 4000 × *g* for 1 min of 40 µL digestion buffer, 40 µL of 0.2% formic acid and finally 35 µL 50% acetonitrile, 0.2% formic acid. Samples were dried under vacuum and stored at –20°C until further use.

Dried peptides were resuspended in 0.1% aqueous formic acid, loaded onto Evotip Pure tips (Evosep Bios) and subjected to LC‐MS/MS analysis using a Exploris 480 Mass Spectrometer (Thermo Fisher Scientific) fitted with an Evosep One (EV 1000, Evosep Bios). Peptides were resolved using a Performance Column 30 SPD (150 µm × 15 cm, 1.5 µm, EV1137, Evosep Bios) kept at 40°C fitted with a stainless‐steel emitter (30 µm, EV1086, Evosep Bios) using the 30 SPD method. Buffer A was 0.1% formic acid in water and buffer B was acetonitrile, 0.1% formic acid.

The mass spectrometer was operated in DIA mode. MS1 scans were acquired in centroid mode at a resolution of 120,000 FWHM (at 200 m/z), a scan ranges from 350 to 1500 m/z, AGC target set to standard and maximum ion injection time mode set to Auto. MS2 scans were acquired in centroid mode at a resolution of 15,000 FWHM (at 200 m/z), precursor mass range of 400–900 m/z, quadrupole isolation window of 12 m/z without window overlap, a defined first mass of 120 m/z, normalised AGC target set to 3000% and maximum injection time mode set to Auto. Peptides were fragmented by HCD (higher‐energy collisional dissociation) with collision energy set to 28% and one microscan was acquired for each spectrum.

The acquired raw‐files were searched using the Spectronaut (Biognosys v19.0) directDIA workflow against a Homo sapiens database (consisting of 20,360 protein sequences downloaded from UniProt on 2022/02/22) and 392 commonly observed contaminants. Default settings were used. Raw MS data are available on ProteomeXchange with the identifier PXD063496.

The raw Spectronaut output data was imported into R (R v. 4.3.2, Bioconductor v. 3.18) and converted into a QFeature object for an integrated analysis of feature to protein level measurements. This analysis included data filtering, aggregation and imputation steps (Gatto and Vanderaa 2024). First, the data was filtered to only include measurement calls with a protein group *q*‐value < 0.01. Next, features were defined by the unique combination of peptide sequence, precursor charge, fragment ion and product charge. Each feature required at least three measurements across the entire data set. Features were aggregated to peptide level by summing all feature intensities for a given peptide per sample. Spectronaut reports two types of missed measurements which require different handling during data aggregation. Intensity values below 1 were treated as measurements below detection limit and were retained at the aggregated level if all feature intensities of a given peptide were below 1 in that sample. Unmeasured features (‘NA’ values) were retained at the peptide level if all feature intensities for a given peptide in that sample were missing. A next filtering step excluded sample‐specific peptides, that is, peptides which were only present in a single biological replicate of one sample group as defined by source (synovial fluid, synovial fibroblasts), fraction (flowthrough, eluate) and disease state. Peptide intensities were then log_2_‐transformed with a pseudo‐count of 1. Peptides with an intensity below detection limit (‘0’ values) were imputed by a Min‐Prob strategy, which samples values from a Gaussian distribution with mean set on low intensity values in each sample. Imputation was done with the Qfeatures function impute and method = ‘MinProb’ and otherwise default options. All ‘NA’ peptide intensities were retained after imputation. In the next step, peptides were aggregated to protein level by a median‐polish strategy using the Qfeatures function aggregateFeatures and as method the MsCoreUtils function medianPolish (Rainer et al. 2022). The imputed protein levels still contained NA values for proteins unmeasured in a given sample. If protein measurements are missing across all samples of a given sample group they are most likely specifically absent in this condition. These cases were therefore treated by an additional MinProb‐based imputation. All remaining NA values were retained in the down‐stream differential analysis, as they can be handled by limma (Ritchie et al. 2015). In a last step, protein intensities per sample were median centered using the QFeatures function normalise and method ‘diff.median’. Principal component analysis (PCA) was performed with package pcaMethods using the method pca, which allowed the treatment of missing data with the nipalsPca algorithm (Stacklies et al. 2007).

For differential expression analysis, the QFeature object was converted into an ExpressionSet object using the normalised protein level data. Cell surface protein scores for each protein were computed using SurfaceGenie (https://gundrylab.shinyapps.io/surfacegenie/) (Waas et al. 2020). Differential expression analysis was based on the limma package (Ritchie et al. 2015). Briefly, a linear model was fit based on the four experimental groups built by crossing source (synovial fluid, synovial fibroblasts) and fraction (flowthrough, eluate). The patient effect was taken into account by estimating sample correlations within patient samples using the duplicateCorrelation function of limma. Functions lmFit and eBayes were used to test specific contrasts such as eluate versus flowthrough in synovial fluid samples. Significance was evaluated based on FDR‐corrected *p* values.

The fraction of missing values (‘NA’) at the protein level was computed before imputation and on a per sample group basis. For the differential analysis of synovial fluid samples, we averaged the fraction missing across samples of different disease state. For example, zero fraction missing (FMiss = 0) means the protein was measured in all samples of a given sample group, while FMiss = 1 implies the protein was unmeasured in the given sample group as defined by fraction and source (Table S2A). Proteins completely missing in synovial fluid samples (*n* = 573) were excluded from the SF‐EV differential analysis report. Proteins from synovial fluid samples (*n* = 3306) were used for differential expression analysis (Table S3). Processed MS data and analysis scripts are available from https://doi.org/10.5281/zenodo.15227820.

### 2.16 Data Download and Processing of Publicly Available Datasets

Publicly available datasets (online repository, web resources) were retrieved from The Human Protein Atlas (HPA) (Digre and Lindskog 2023), Uniprot (UniProt 2025), PanglaoDB (Franzen et al. 2019), ExoCarta (Simpson et al. 2012), GeDiPNet (Kundu et al. 2023), Tabula Sapiens (Tabula Sapiens et al. 2022) and the Broad Institute Single Cell Portal (SCP) using the Accelerating Medicines Partnership (AMP) Phase I dataset for rheumatoid arthritis synovial tissue (dataset 1: Rheumatoid Arthritis [Tarhan et al. 2023; Zhang et al. 2019]). Where applicable, published data sets were obtained either from journal supporting information or provided from official depositories associated with the publication. To harmonise identifiers across sources, all downloaded datasets including gene/protein entries were mapped to HPA‐annotated symbols using the reference set of 20,082 proteins (annotation status: OCT 2024). Entries in downloaded datasets not represented in the HPA annotation were excluded from downstream analyses.

### 2.17 Functional Annotation and Protein Family Inspection

Distribution of SF‐EV proteins into the three cell compartment classes ‘Intracellular, Secreted and Membrane’ was based on the HPA classification. To characterise the functional composition and asses a protein family classification of the SF‐EV proteome (including protein class, molecular function, biological process and cellular composition), we performed Gene Ontology enrichment and KEGG pathway analysis using g:Profiler (Kolberg et al. 2023) (update 2023) an interoperable web server for functional enrichment analysis and gene identifier mapping. To link translational relevance to the SF‐EV proteins we intersected the union of 3306 proteins with curated lists of arthritis‐relevant gene candidates (derived from the HPA and GeDiPNet database) to highlight potential biomarker candidates within the SF‐EV proteome.

### 2.18 Surfaceome Scoring and Marker Prioritisation

To assist cell type‐specific marker identification present on the SF‐EV surface and to prioritise potential targets for future immunocapture, we used SurfaceGenie (Waas et al. 2020), a web‐based application, which assigns a cell surface protein consensus (SPC) score based on curated surfaceome evidence, thus predicting the likeliness of a protein to be localised to the cell surface.

### 2.19 Cell Type Signatures

Curated cell type signatures (CTS) representing 10 synovium‐associated cell types were generated from gene/protein expression resources in HPA (Digre and Lindskog 2023) and PanglaoDB (Franzen et al. 2019) (Table S4). The CTS panel comprised fibroblasts, muscle cells, a composite mesenchymal/stromal set (osteoblasts, osteocytes, chondrocytes, adipocytes), dendritic cells, neutrophils, macrophages, monocytes, T‐cells, NK cells, B‐cells and endothelial cells.

### 2.20 Data Analysis, Visualisation and Statistics

Data were analysed and visualised as bar plots or box and whiskers plots using GraphPad Prism version 10.2.1 for MacOS (GraphPad Software Inc) and Microsoft Excel. Dataset overlaps and Venn diagrams were generated using InteractiVenn (Heberle et al. 2015). Biorender was used to design schematic overviews. Comparisons between samples were performed using student's paired or unpaired *t*‐test and presented as either individual values, bar graphs or box plots representing the interquartile range (IQR) and median, with whiskers depicting the 5th and 95th percentile. *p* values < 0.05 were considered statistically significant. Data was processed in GraphPad Prism.

## 3 Results

### 3.1 Exploratory Pre‐Screening Detects Stromal‐ and Myeloid‐Associated Markers in Synovial Fluid EVs From Arthritis Patients

Understanding the cellular origins and surface marker profiles of extracellular vesicles (EVs) in synovial fluid is critical for their use as minimally invasive biomarkers in arthritis. Synovial fluid is in direct contact with inflamed synovial tissue and contains a heterogenous pool of EVs derived from tissue‐resident and infiltrating cell populations, including synovial fibroblasts, immune cells and endothelial cells among others (Figure 1a). To establish whether candidate markers relevant for subsequent targeted EV enrichment were detectable in synovial fluid of arthritis patients, we first performed an exploratory pre‐screening of bulk synovial fluid (SF)‐EV preparations.

**FIGURE 1:**
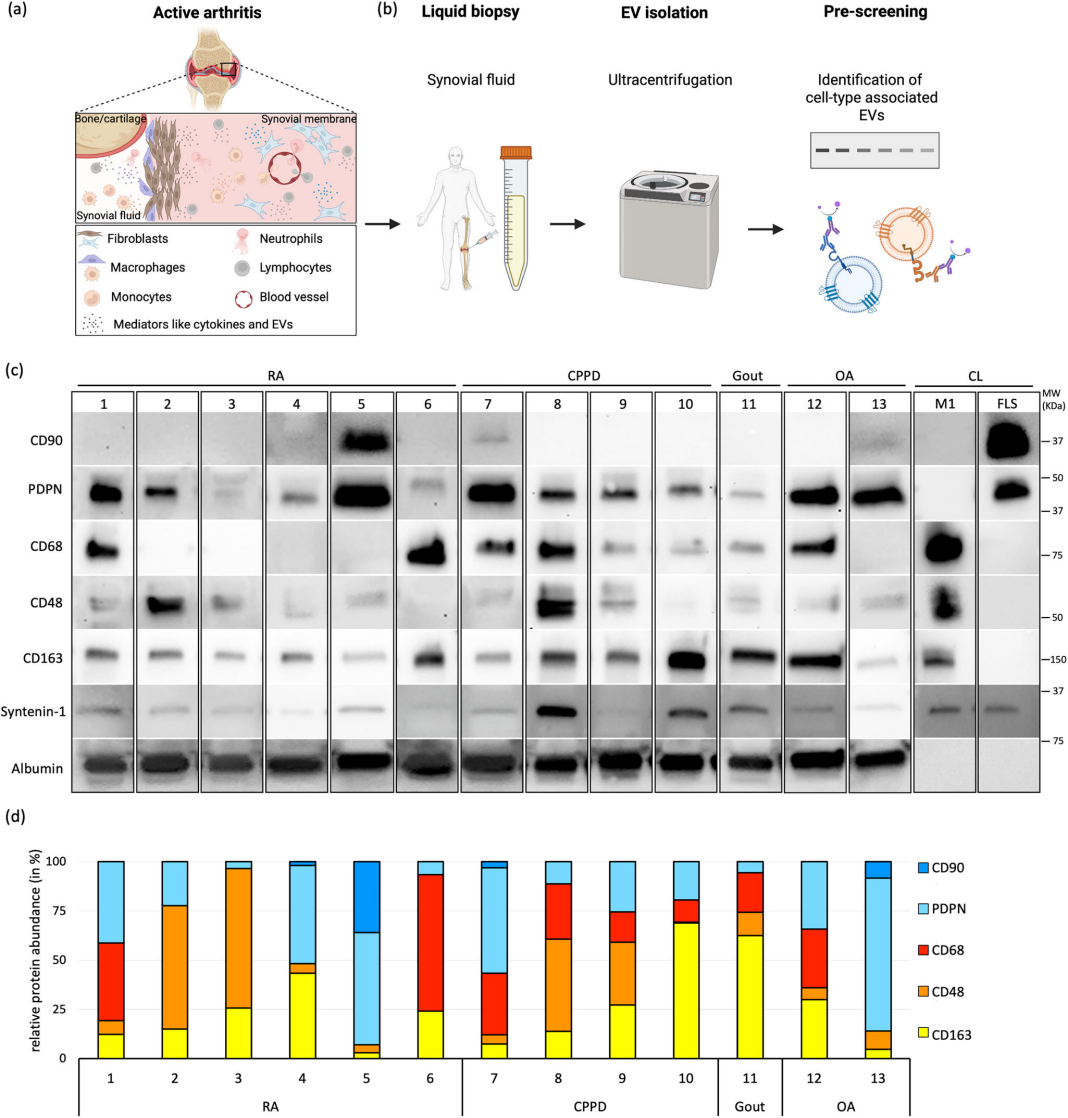
Ultracentrifugation‐based exploratory pre‐screening reveals heterogeneous stromal and myeloid surface‐marker patterns across synovial fluid EV isolates from arthritis patients. (a) Schematic overview of synovial tissue and major cell populations present in active arthritis. (b) Workflow for ultracentrifugation (UC)‐based exploratory pre‐screening of synovial fluid (SF)‐EV marker profiles. (c) Western blot analysis of UC‐derived bulk SF‐EV preparations from patients with different forms of arthritis (total *n* = 13; RA *n* = 6, CPPD *n* = 4, Gout *n* = 1, OA *n* = 2) loaded by equal total protein amounts (50 µg per lane) and probed for CD90, PDPN, CD68, CD48, CD163, syntenin‐1 and human serum albumin. Cell lysates (CL; 2.5 µg per lane) from cultured synovial fibroblasts (FLS) and M1‐like macrophages (M1) were included as positive controls for antibody reactivity and to confirm cell‐type‐association. For each patient sample, the marker panels shown were derived from the same immunoblot. (d) Relative distribution of selected markers across donor‐derived SF‐EV preparations. Densitometric quantification of marker signals normalised to the corresponding syntenin‐1 signal, shown as the percentage contribution of normalised intensity across donor samples. CPPD, calcium pyrophosphate deposition (CPPD) arthritis; OA, osteoarthritis; RA, rheumatoid arthritis.

To differentiate distinct EV populations in arthritic synovial fluid, we selected a panel of surface‐associated protein markers linked to synovial stromal and myeloid cell compartments, reported to represent abundant pathological cell populations in arthritic synovium (Zhang, Jonsson, et al. 2023). These included CD90/THY1 and Podoplanin (PDPN), both associated with pathogenic synovial fibroblasts (Mizoguchi et al. 2018) and previously detected in SF‐EVs by mass‐spectrometry (Foers et al. 2020), as well as CD68, CD48 and CD163, linked to different macrophage populations in inflamed synovial tissue of RA patients (Alivernini et al. 2020; Amin et al. 2017). The marker selection was additionally guided by surface accessibility, membrane topology and reported detection in synovial fluid or tissue proteomic studies.

To assess detectability of the selected markers in arthritic synovial fluid samples and obtain an initial view of inter‐donor variation equal volumes of cell‐free synovial fluid from 13 patients with RA, OA or other forms of arthritis were processed using a basic ultracentrifugation (UC)‐based bulk EV isolation (Figure 1b). Western blot analysis of equal protein amounts from the resulting UC‐EV pellets revealed heterogenous marker profiles across patient samples (Figure 1c). PDPN, a pan‐fibroblast marker, was detected across all samples, whereas CD90 varied substantially between donors and was not detectable in a subset of the bulk EV preparations. Among myeloid‐associated markers, CD163 was consistently detected, indicative for tissue‐resident synovial macrophages, while CD68 and CD48 showed more variable signal intensities. This apparent inter‐sample heterogeneity among the SF‐EV preparations extended beyond marker expression and was accompanied by visible differences in physical synovial of the fluid properties, including colour and viscosity, as well as variation in the total protein content recovered in UC‐derived EV pellets per input synovial fluid volume.

At the same time, technical variability potentially introduced by the UC‐based bulk EV isolation from this complex biofluid may also contribute to in‐between‐sample differences and must be considered when interpreting bulk pre‐screening data. To exclude technical variation associated to the EV isolation workflow we performed a technical reproducibility experiment. To assess technical consistency under same input conditions, one representative synovial fluid sample was processed independently in three parallel EV preparations and analysed side‐by‐side by Western blot for the marker panel. The replicate isolations showed comparable EV marker patterns across all three preparations, supporting reproducible recovery of SF‐EV‐associated proteins from the same donor input and arguing against major isolation‐driven batch effects (Figure S1a). Finally, immunoblot‐based readouts comparing marker abundance require careful normalisation. To account for the different EV‐associated protein contributions to the total protein amount in a given sample, we used syntenin‐1 as an internal EV‐associated reference protein for the quantification of Western Blot signal intensities. Syntenin‐1 is a commonly detected EV‐associated protein marker reported across diverse cellular sources, species and biofluids (Kugeratski et al. 2021). Notably, serum albumin was co‐enriched in all UC‐EV isolates, underlining the substantial fraction of non‐EV background inherent to complex matrices like serum‐enriched synovial fluid and to UC‐based bulk EV isolation workflows. In parallel, syntenin‐1 signal intensities varied across donor samples, indicating differences in the relative contribution of EV‐associated material present within the fixed total protein load (Figure 1c). Normalisation of EV marker PDPN, CD90, CD68, CD48, CD163 signals to syntenin‐1 enabled a more robust comparison of relative marker patterns across the screened samples despite heterogenous background and variable EV content, and identified CD90 as the most variable marker among those analysed (Figure 1d). These exploratory data confirmed the detectability of selected stromal and myeloid markers in bulk SF‐EV preparations from arthritis patients, while indicating marker heterogeneity across samples. This provided the rationale for developing a more selective EV isolation and enrichment strategy to resolve discrete marker‐positive EV subpopulations from complex arthritic synovial fluid.

### 3.2 In Vitro Validation of CD90/PDPN Surface Accessibility and Selective EV Immunocapture in Synovial Fibroblast‐Derived EVs

The lack of specific methods for isolating and characterising distinct EV subpopulations from complex biofluids poses a major challenge in understanding the roles of disease‐relevant EV populations. Most studies investigating EVs are based on conventional bulk EV isolation techniques, which recover a heterogenous population of vesicles, and can therefore obscure insights into discrete subpopulations originating from specific disease‐relevant cell types. To address this limitation and enable the targeted enrichment of stromal‐associated disease‐relevant EV subpopulations from complex synovial fluid in arthritis, we first established the technical feasibility of surface marker‐directed immunocapture coupled to size exclusion chromatography (SEC) and ultrafiltration (UF) in a controlled in vitro system. CD90 and PDPN were selected as candidate stromal‐associated synovial target markers, based on their detection in bulk SF‐EV samples during the UC‐based pre‐screening (compare Figure 1) and their documented expression by synovial fibroblasts populations reported in recent synovial tissue profiling studies (Mizoguchi et al. 2018; Croft et al. 2019). CD90 encodes a GPI‐anchored membrane protein located in the outer leaflet of lipid rafts on synovial fibroblasts, whereas PDPN is a type I transmembrane sialomucin‐like glycoprotein broadly associated with activated stromal populations in arthritic synovium. Both proteins are heavily glycosylated and contribute to defining cellular phenotypes through protein‐protein interactions, particularly in cancer and immune responses (Yang et al. 2020; Zhang et al. 2022). In the present study, CD90 served as the principal proof‐of‐concept target for stromal‐associated EV enrichment, while PDPN was included as an additional broad fibroblast‐associated surface marker for validation of the immunocapture approach.

To validate CD90 and PDPN as targetable stromal EV surface markers and develop a selective EV subpopulation isolation strategy, we first used a controlled in vitro cell culture system. Synovial fibroblasts isolated from arthritis patient synovial tissue biopsies were cultured ex vivo, and their conditioned medium (CM) was processed to isolate small EVs (Figure 2a). Because CM was harvested in large volumes, an initial ultrafiltration‐based concentration step was required before SEC‐UF enrichment to generate a suitable input for downstream EV characterisation and immunocapture. The SEC‐UF‐enriched EVs were characterised by transmission electron microscopy (TEM) and nanoparticle tracking analysis (NTA) confirming a typical small EV morphology and size distribution (30–130 nm), together with particle concentrations in the range of 1–2 × 10^10^ particles/mL. Moreover, positive single‐vesicle surface staining using nFCM under non‐fix, non‐permeabilisation conditions demonstrated the localisation of CD90 and PDPN on the EV surface, thereby indicating antibody‐recognizable epitopes of membrane‐localised CD90 and PDPN exposed on intact EVs (Figure 2b,c; Figure S2). Western blot analysis using representative arthritis‐derived synovial fibroblast EV preparations as validation samples further confirmed the presence of CD90 and PDPN expression on fibroblast‐secreted EVs, as both proteins were enriched in the membrane protein fractions of these EVs. CD90 and PDPN were detected alongside canonical EV markers CD63, CD9, CD81, flotillin‐1, annexin‐1 and syntenin‐1, whereas the EV exclusion marker calnexin was only observed in the corresponding control cell lysates, confirming the purity of the enriched EV preparations (Figure 2d,e). These findings supported the suitability of CD90 and PDPN as accessible target markers suitable for immunocapture‐based EV enrichment strategies.

**FIGURE 2:**
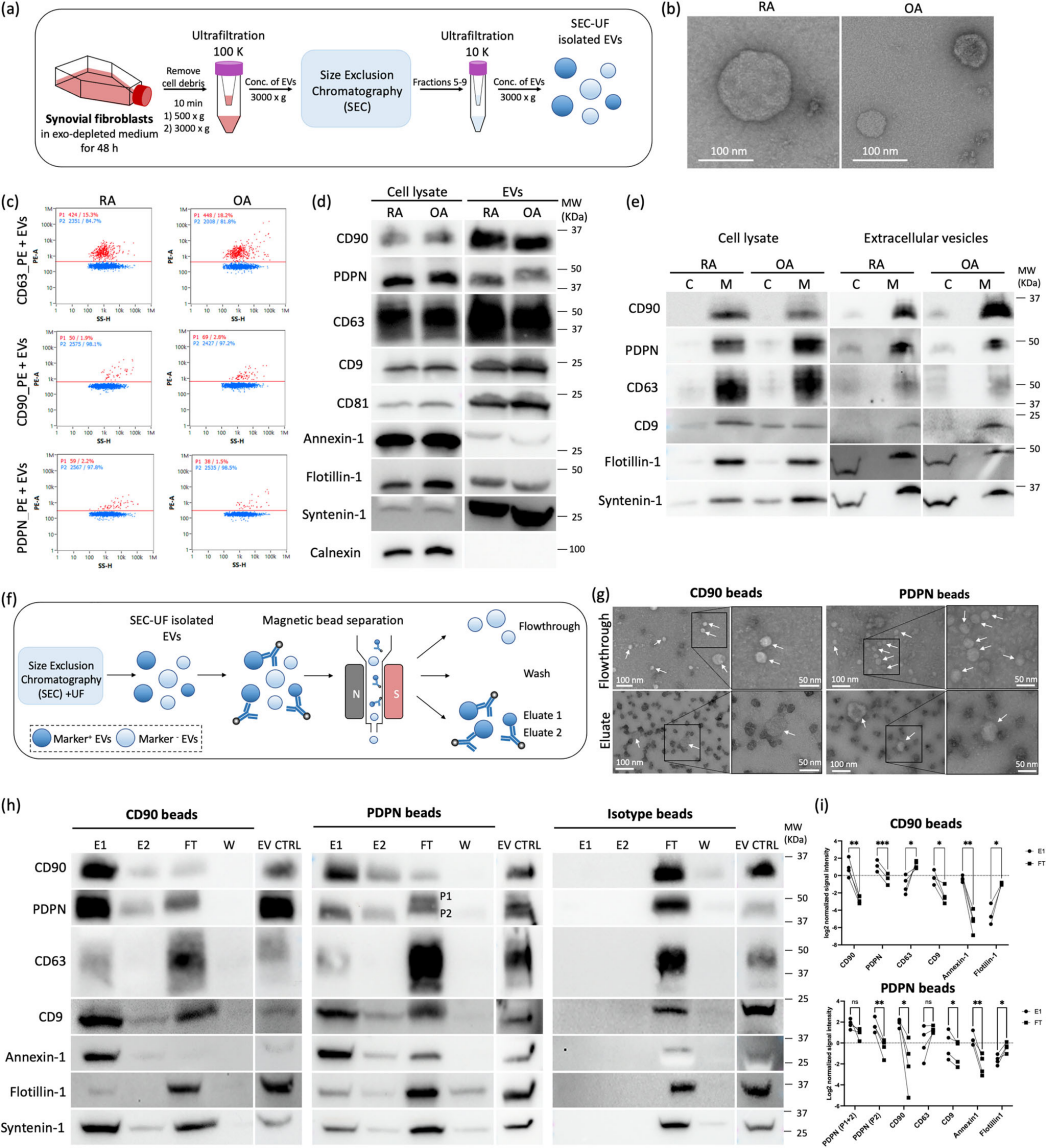
In Vitro validation of CD90‐ and PDPN‐directed immunocapture of synovial fibroblast‐derived EVs following SEC‐UF enrichment. (a) Schematic of the size exclusion chromatography‐ultrafiltration (SEC‐UF) workflow used to enrich EVs from conditioned medium (CM) of cultured synovial fibroblasts. (b) Representative transmission electron microscopy (TEM) images of SEC‐UF‐enriched EVs from two arthritis‐derived synovial fibroblasts (RA and OA donor). (c) Nano‐flow cytometry (nFCM) single‐vesicle surface staining of synovial fibroblast‐derived EVs for CD63, CD90 and PDPN under non‐fixed, non‐permeabilised conditions. Data are shown for two representative arthritis‐derived synovial fibroblast EV preparations (RA and OA). (d) Representative Western blot analysis of SEC‐UF‐enriched EVs and corresponding cell lysates (CL) from two arthritis‐derived synovial fibroblasts (RA and OA). Blots were probed for canonical EV markers (CD63, CD9, CD81, syntenin‐1, annexin‐1, flotillin‐1), fibroblast‐associated markers (CD90, PDPN) and the EV exclusion marker calnexin. (e) Representative Western blot analysis of cytosolic (C) and membrane‐enriched (M) protein fractions from cell and SEC‐UF‐enriched EV lysates of two arthritis‐derived synovial fibroblasts preparations (RA and OA). Blots were probed for CD90, PDPN and for canonical EV markers (CD63, CD9, syntenin‐1, annexin‐1, flotillin‐1). (f) Schematic of magnetic bead‐based immunocapture of SEC‐UF‐enriched EVs using nanosised beads coupled to anti‐CD90 or anti‐PDPN antibodies, or matched isotype control antibodies. Marker‐negative or non‐captured EVs were collected in the flowthrough (FT), followed by collection of a wash fraction (W). Bead‐bound marker‐positive EVs were recovered in the first eluate (E1), with a second eluate (E2) collected to assess residual carryover and elution efficiency. (g) Representative TEM images of E and FT fractions following CD90‐ or PDPN‐directed immunocapture; arrows indicate EVs. (h) Representative Western blot analysis of EV fractions obtained after CD90‐ and PDPN‐directed immunocapture, including the EV input control (EV CTRL), fractions E1‐E2, W, FT and corresponding isotype‐bead control. Blots were probed for CD90, PDPN and canonical EV markers (CD63, CD9, syntenin‐1, annexin‐1, flotillin‐1). (i) Densitometric quantification of immunocapture outcomes from Western blot analyses of *n* = 3 independent experiments using SEC‐UF‐enriched EVs from synovial fibroblasts of different arthritis patients, shown as log_2_ relative density ratios for proteins of interest after normalisation to syntenin‐1. Paired E‐versus‐FT comparisons were assessed by paired Student's *t*‐test (ns, not significant; *p < 0.05; **p < 0.005; ***p < 0.001). Lines connect matched E‐FT pairs. P1 and P2 denote two PDPN signal populations resolved by immunoblot.

We next applied prototype nanosised (50 nm) magnetic beads functionalised with anti‐CD90 or anti‐PDPN antibodies (Miltenyi) to SEC‐UF‐enriched synovial fibroblast‐derived EV preparations (Figure 2f). Following magnetic separation, TEM and Western blot analysis of the eluate (E) and flowthrough (FT) fractions confirmed selective enrichment of CD90^+^ and PDPN^+^ EV subpopulations (Figure 2g). CD90^+^ EVs in fraction E were enriched for PDPN, annexin‐1 and CD9, while CD63 and flotillin‐1 were more abundant in the CD90^−^ FT fraction, highlighting distinct marker signatures between EV subtypes (Figure 2h,i). Syntenin‐1 signals remained comparable across all fractions, indicating comparable EV‐associated proteins loads between the total protein‐normalised samples. Isotype bead controls showed no detectable EV capture, supporting antibody‐dependent enrichment under these experimental conditions.

Interestingly, PDPN‐directed immunocapture revealed additional heterogeneity among PDPN^+^ EV populations. A CD90^+^/PDPN^+^ EV fraction was enriched in E, whereas an additional PDPN^+^ EV subset remained detectable in the FT fraction, which showed a distinct higher molecular weight pattern (Figure 2h, Figure S3a). A similar PDPN isoform was observed in the CD90^−^ EV fraction (FT) following the CD90‐immunocapture. These findings suggest that PDPN‐associated EV populations may be heterogenous, potentially displaying discrete chemically‐modified PDPN isoforms which are selectively co‐expressed with CD90, and not accessible via the anti‐PDPN antibody coupled to the used PDPN beads.

To further test selectivity and robustness of our technical approach under conditions of increased sample complexity, we performed a panel of spike‐in experiments using defined EV mixtures. In one setup, CD90^+^ fibroblast‐derived EVs were mixed 1:1 with CD68^+^ macrophage‐derived EVs generated from differentiated THP‐1 cells. Following CD90‐directed immunocapture, CD90^+^ EVs were selectively retrieved in the E fraction, while CD68^+^ EVs remained confined to the FT (Figure 3a,b). To translate our workflow to complex biofluid conditions, we performed a second experiment. EVs derived from CD90^+^ synovial fibroblasts were spiked into a native synovial fluid sample that lacked a detectable endogenous CD90 signal in the UC pre‐screen. This design allowed the selective recovery of the introduced CD90^+^ EV population to be monitored against the complex synovial fluid background. Western blot analysis demonstrated that the CD90‐directed immunocapture retrieved the spiked CD90^+^ EVs with reduced, but still detectable, efficiency, indicating that the approach is applicable to complex biofluid matrices (Figure 3c,d). Collectively, these experiments establish that CD90‐directed immunocapture can selectively enrich CD90^+^ EVs from controlled EV mixtures and remains technically feasible in a synovial fluid background, thereby supporting a subsequent application of the refined workflow to patient‐derived synovial fluid.

**FIGURE 3:**
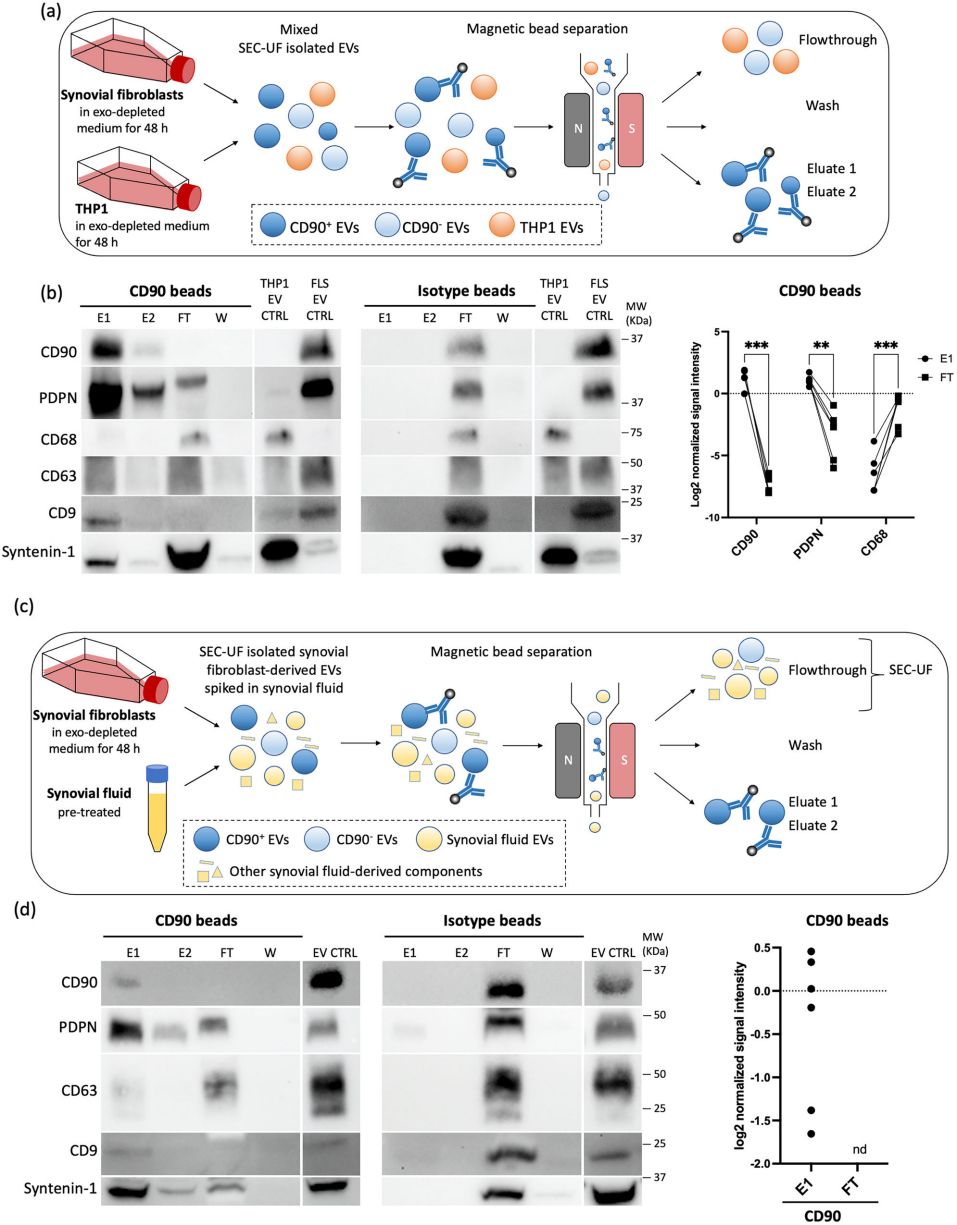
CD90‐directed immunocapture selectively retrieves CD90+ EVs from mixed EV preparations and synovial fluid‐spiked samples. (a) Schematic of immunocapture from a defined mixed EV preparation generated by combining SEC‐UF‐enriched EVs from synovial fibroblasts and THP‐1 macrophages at a 1:1 ratio. CD90^+^ EVs are captured and eluted (E1, E2), while unbound EVs are collected as flowthrough (FT) and wash (W). (b) Representative Western blot analysis of E and FT fractions from the CD90‐directed immunocapture using the mixed EV preparation, probed for CD90, PDPN, CD68 and for canonical EV markers CD63, CD9 and syntenin‐1. An isotype‐bead control was included to assess capture specificity. EV input controls (bulk SEC‐UF‐enriched EVs prior to immunocapture) from each source are shown. Densitometric quantification of CD90‐directed immunocapture outcomes from Western blot analyses of *n* = 3 independent experiments using SEC‐UF‐enriched EVs from synovial fibroblasts of different arthritis patients, shown as log_2_ relative density ratios for CD90, PDPN and CD68 after normalisation to syntenin‐1. Paired E‐versus‐FT comparisons were assessed by paired Student's *t*‐test (**p < 0.005; ***p < 0.001). Lines connect matched E‐FT pairs. (c) Schematic of CD90‐directed immunocapture following spiking of synovial fibroblast‐derived SEC‐UF‐enriched EVs into a pre‐treated synovial fluid sample lacking detectable endogenous CD90 signal. The fractions E1, E2, W and FT were collected; unbound EVs in FT were subsequently purified and enriched by SEC‐UF as indicated. (d) Representative Western blot analysis of E1 and E2, FT and W fractions from synovial‐fluid spiked‐in experiments, probed for CD90, PDPN and for canonical EV markers CD63, CD9 and syntenin‐1, with an isotype‐bead control and EV input control (spiked SEC‐UF EV population). Densitometric quantification of immunocapture outcomes shown as log_2_ relative density ratios for CD90 signal intensity normalised to syntenin‐1. No statistical assessment was performed from the paired E1 versus FT, as no CD90 signal was detectable in the FT fraction (nd, not detected).

### 3.3 SEC‐UF Pre‐Enrichment Supports CD90‐ and PDPN‐Directed Immunocapture of EV Subpopulations From Arthritic Synovial Fluid

Building on the demonstration that the SEC‐UF‐coupled immunocapture principle is technically feasible for the detection of CD90^+^ EVs in CM, we next adapted the workflow to synovial fluid samples from arthritis patients. Isolating endogenous EV populations from synovial fluid presents significant technical challenges due to its molecular composition and limited volume availability. In contrast to cell culture‐derived CM, synovial fluid represents a highly complex biofluid, characterised by variable viscosity, extracellular matrix (ECM) components, serum ultrafiltrate, and additional soluble background material that can interfere with EV recovery and downstream‐affinity‐based separation (Foers et al. 2018). To overcome these matrix‐specific constraints, the SEC‐UF protocol was preceded by several synovial fluid pre‐treatment steps according to recommendations of the Synovial Fluid Task Force MISEV guidelines (Welsh et al. 2024). This treatment scheme included hyaluronidase digestion, DNAse treatment, addition of protease/phosphatase inhibitors, debris removal by centrifugation and filtration, and adequate dilution to support optimal performance during SEC‐UF EV isolation (Figure 4a). Following these preparatory steps, TEM analysis confirmed the recovery of intact, round‐shaped EVs in SEC‐UF‐enriched preparations, with reduced visually apparent background compared with pre‐treated synovial fluid without enrichment (Figure 4b).

**FIGURE 4:**
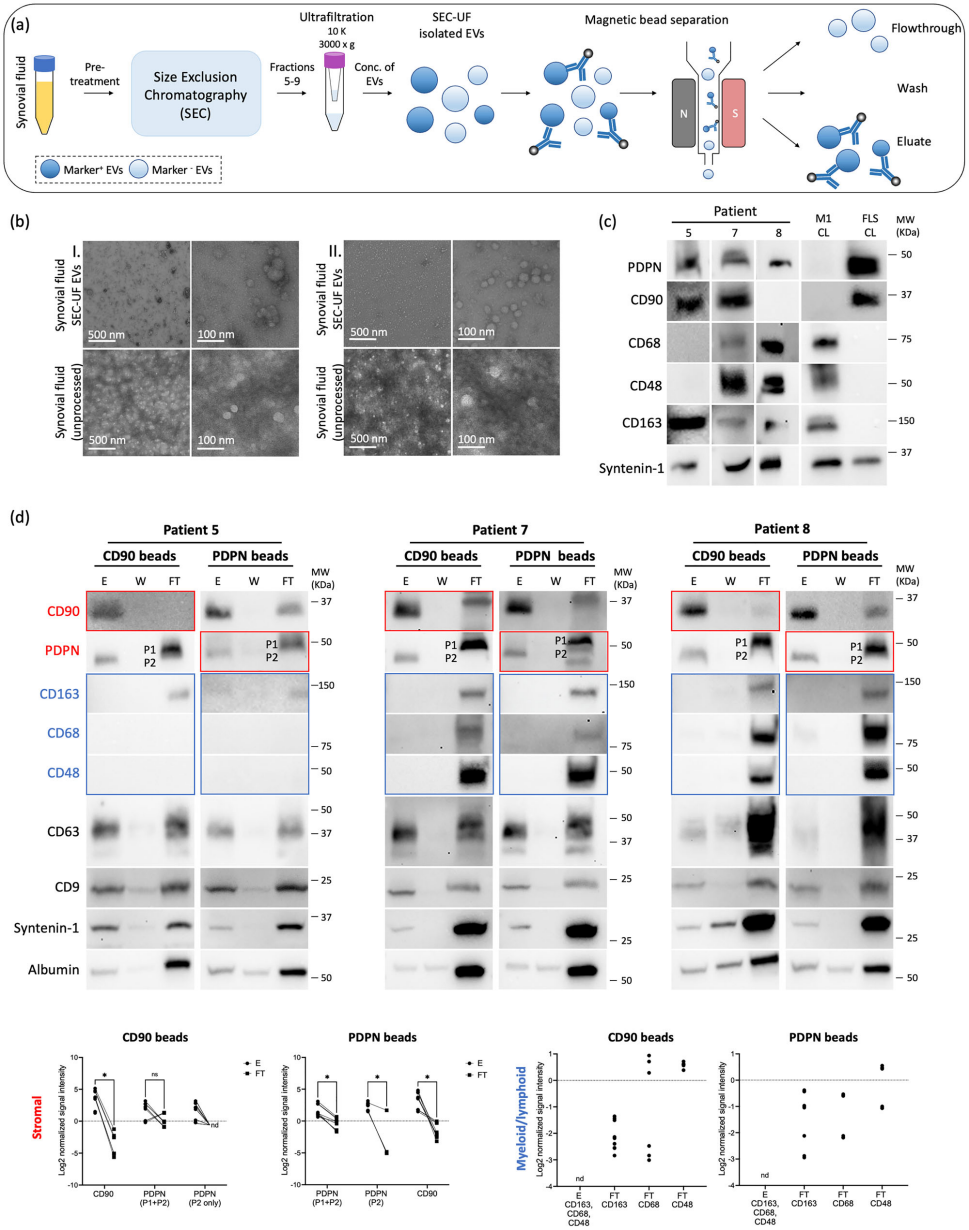
SEC‐UF enrichment enables CD90‐ and PDPN‐directed immunocapture of stromal‐associated EV populations from arthritic synovial fluid. (a) Schematic of the synovial fluid pre‐treatment and SEC‐UF EV enrichment workflow, followed by magnetic bead‐based immunocapture using nanosised antibody‐coupled beads. (b) Representative transmission electron microscopy (TEM) images of two independent synovial fluid preparations with and without SEC‐UF processing, illustrating the reduction of visually apparent background material and recovery of EVs following the enrichment workflow. (c) Western blot analysis of SEC‐UF‐enriched SF‐EV preparations from three arthritis patients (Patients 5, 7 and 8), probed for stromal‐associated markers (CD90, PDPN), selected myeloid/immune‐associated markers (CD68, CD48, CD163) and syntenin‐1. Cell lysates (CL; 2.5 µg per lane) from cultured synovial fibroblasts and M1‐like macrophages were included as positive controls for antibody reactivity and to confirm cell‐type‐association. For each patient sample, all marker panels shown were derived from the same immunoblot. (d) Western blot analysis and densitometric quantification of EV fractions obtained after immunocapture in SEC‐UF‐enriched SF‐EVs using anti‐CD90 or anti‐PDPN beads, shown for patients 5, 7 and 8. Eluate (E), flowthrough (FT) and wash (W) fractions are displayed on the same immunoblot; total recovered protein material from each fraction was analysed. Stromal‐associated signals (CD90, PDPN; red frames) and selected myeloid/immune‐associated signals (CD163, CD68, CD48; blue frames) are indicated, together with probed canonical EV markers (CD63, CD9, syntenin‐1) and human serum albumin. For quantification, band intensities were normalised to syntenin‐1 and expressed as log_2_ relative density ratios. Paired E‐versus‐FT comparisons for CD90 and the two PDPN isoforms (P1 and P2) were assessed by paired Student's *t*‐test (ns, not significant; *p < 0.05; **p < 0.005; ***p < 0.001). Lines connect matched E–FT pairs. No statistical assessment was performed for the selected myeloid/immune‐associated markers because CD68, CD48 and CD163 signals were not detectable in the corresponding E fractions (nd, not detected).

To validate the refined SEC‐UF‐coupled immunocapture workflow in patient‐derived samples, we analysed three pre‐screened synovial fluid specimens with distinct CD90/CD68 marker profiles, as shown in the initial UC‐based screen and in the corresponding SEC‐UF‐enriched bulk SF‐EV preparations (Patients 5, 7 and 8; Figure 4c, Figure S3b). The SEC‐UF‐enriched SF‐EVs were subjected to magnetic bead separation using anti‐CD90 or anti‐PDPN beads. TEM imaging showed intact EVs bound to beads in the E fraction, while bead‐unbound, free EVs accumulated in the corresponding FT fraction (Figure S3c). Western blot analysis and densitometric quantification showed successful recovery of CD90^+^ EVs in E across all three specimens, including Patient 8, in which CD90 was initially not detectable in the corresponding bulk EV preparation (Figure 4d). These findings indicated that targeted immunocapture is able to enrich a low‐abundance EV subpopulation otherwise remained below detection within heterogeneous bulk EV preparations. Notably, the enriched CD90^+^ EVs in fraction E co‐enriched PDPN and annexin‐1, while the selected macrophage‐associated markers CD68, CD48 and CD163 were not detectable. In contrast, the CD90^−^ FT fraction was enriched for myeloid markers, supporting the specificity of the anti‐CD90 beads in separating a stromal‐associated EV fraction from a broader non‐captured SF‐EV pool containing selected myeloid cell type‐associated EVs. Furthermore, a distinct PDPN isoform (P1) was also observed in the FT fraction, consistent with the PDPN pattern identified in synovial fibroblast‐derived EVs. Elevated syntenin‐1 and albumin signals in the FT fraction reflected the non‐captured SF‐EV pool and residual soluble protein background remaining following the SEC‐UF processing. Similar fractionation patterns were obtained following PDPN‐directed immunocapture (Figure 4d).

To assess whether the established immunocapture workflow could be performed without prior SEC‐UF enrichment, we next applied the anti‐CD90 and anti‐PDPN bead‐based approach directly to concentrated cell culture CM and to pre‐treated synovial fluid. Direct immunocapture from concentrated synovial fibroblast CM resulted in efficient enrichment of CD90^+^ EV and PDPN^+^ EV subpopulations in the E fractions, closely reflecting the results obtained with SEC‐UF‐enriched EV inputs (Figure S4a). In contrast, direct immunocapture from pre‐treated synovial fluid produced more variable results in two patient samples tested (Figure S4b,d). In Patient 8, direct CD90^+^ EV recovery broadly recapitulated the SEC‐UF‐coupled result. However, in Patient 5, CD90^+^ EV‐associated signal was not efficiently recovered in the E fraction, and macrophage‐associated markers were incompletely removed. In addition, albumin signals in these fractions were more pronounced than in SEC‐UF pre‐enriched fractions (see Figure 4d, Figure S4d), indicating that the increased impurity of the direct input material, without preceding SEC‐UF processing, resulted in a substantial portion of soluble protein background remaining in the sample. These results show that while direct immunocapture from cell culture CM is feasible, direct immunocapture from pre‐treated synovial fluid can be impeded by the complex biofluid matrix yielding less predictable outcomes. Accordingly, the combination of SEC‐UF enrichment with immunocapture improves the reliability and robustness of targeted EV recovery under the tested conditions. To further assess technical consistency of the refined workflow, one pre‐treated synovial fluid sample was divided into three aliquots and processed independently through SEC‐UF enrichment. In addition, two aliquots of the same sample were independently processed in a one‐month interval, to test for day‐to‐day batch effects. Western blot analysis of the resulting EV preparations showed comparable marker patterns across the replicate isolations, supporting reproducible recovery of SF‐EV proteins under the same input conditions (Figure S1b,c).

Collectively, these data show that synovial fluid requires sample‐adapted preparation before affinity‐based EV separation and that SEC‐UF pre‐enrichment facilitates subsequent CD90‐ and PDPN‐directed immunocapture from this complex biofluid. Thus, the refined workflow provides a fit‐for‐purpose approach for reliably recovering EV subpopulations from complex biofluids, and serves as a critical preparatory step for sensitive downstream analyses like quantitative proteomics.

### 3.4 LC‐MS/MS Profiling Establishes Broad EV‐Associated Proteome Coverage and Biological Context of SF‐EVs in Arthritis

Mass spectrometry (MS)‐based proteomics provides a sensitive approach for assessing the molecular composition of EV preparations (Cross et al. 2024) and for determining whether sufficient proteome depth is achieved for downstream comparison of EV subpopulations. To evaluate the proteomic coverage obtained with our SEC‐UF‐based workflow, we performed DIA LC‐MS/MS profiling of SEC‐UF‐enriched SF‐EVs from arthritis patients. In accordance with previous studies profiling SF‐EVs, SEC‐UF EV preparations were used as input for LC‐MS/MS analysis (Foers et al. 2020). This approach reduced, but not completely eliminated, the influence of the wide protein concentration range present in unfractionated biological samples (Dayon et al. 2022), where high‐abundance plasma proteins from serum infiltrates and ECM components can mask less abundant EV‐associated proteins, potentially obscuring subtle disease‐relevant features encoded in the EV proteome (Foers et al. 2018).

Across the 11 patient‐derived SF‐EV preparations, LC‐MS/MS analysis identified 3306 proteins (Table S2A). To asses EV‐associated proteome coverage and estimate the contribution of synovial fluid‐associated proteins beyond plasma‐attributed components, we compared our data with two reference datasets: (i) the ExoCarta database of 7862 human MS‐measured small EV proteins, a publicly accessible curated repository compiling protein/RNA/lipid cargo data identified in small EVs from >1200 conducted studies (Simpson et al. 2012) (Version 6, 2024); and (ii) a set of 551 high‐abundance plasma proteins (concentration >0.1 mg/L assessed in human blood plasma) listed in the Human Protein Atlas database (HPA/PeptideAtlas) (Digre and Lindskog 2023). Of the 3306 proteins identified in the SF‐EV dataset, 2961 overlapped with the ExoCarta‐listed proteins, corresponding to approximately 90% of the total dataset and confirming broad EV‐associated proteome coverage (Figure 5a, Table S2B). In parallel, 405 out of 551 high‐abundance plasma proteins were detected, corresponding to 74% of this reference set and highlighting the expected substantial contribution of plasma ultrafiltrate and soluble protein background to synovial fluid. Notably, 246 proteins in our dataset were not previously reported in ExoCarta. Comparison with the SEC‐processed SF‐EV proteome reported by Foers et al. (2020) identified 200 proteins not previously described in MS‐based SF‐EV datasets, indicating improved detection of low‐abundance proteins potentially associated with SF‐EV populations that were previously not detectable in bulk SF‐EV proteomic surveys.

**FIGURE 5:**
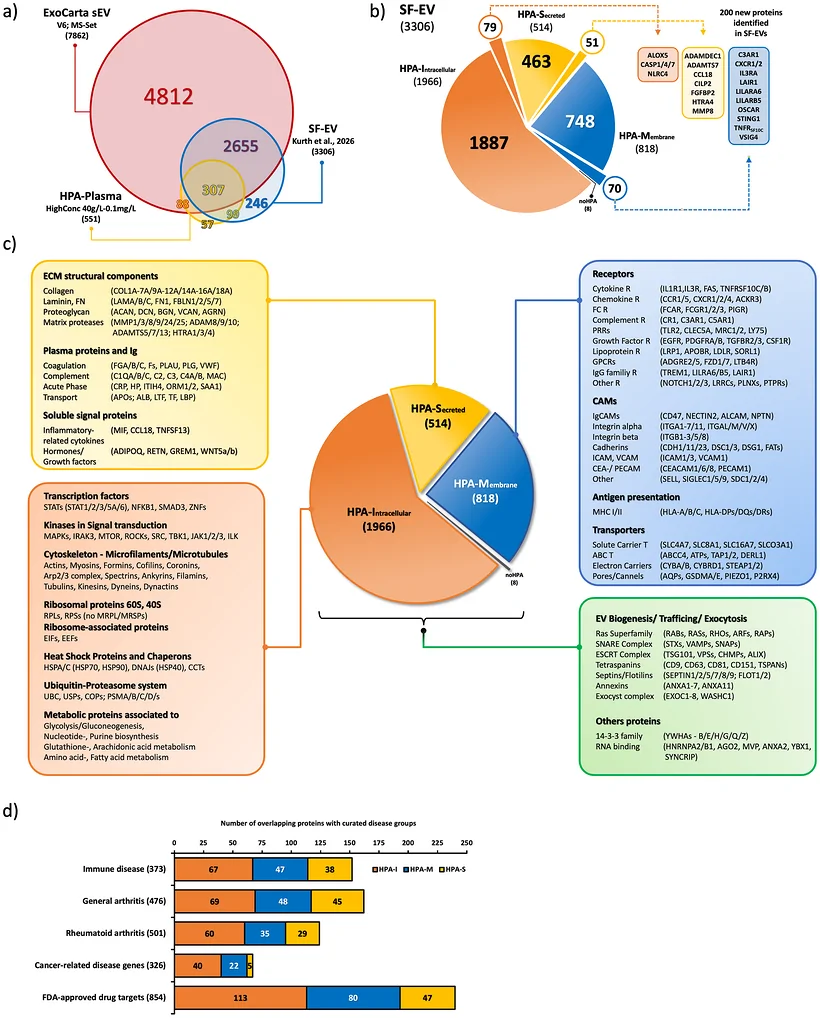
LC‐MS/MS profiling reveals broad EV proteome coverage and biological context of EV preparations from arthritic synovial fluid. (a) Venn diagram showing overlap between the synovial fluid (SF)‐EV proteome identified in this study (3306 proteins, blue circle) and two reference datasets: ExoCarta Version 6, comprising MS‐reported small EV proteins (red circle) and a curated set of high‐abundance plasma proteins from the Human Protein Atlas (HPA, MS‐reported, orange circle). Assigned numbers indicate unique or shared proteins across the compared datasets; numbers in parentheses indicate the total size of each dataset. (b) Distribution of the SF‐EV proteome across three HPA cellular compartment annotations: intracellular (HPA‐I), membrane‐associated (HPA‐M) and secreted (HPA‐S). Numbers in parentheses indicate the total number of SF‐EV proteins assigned to each compartment. The subset of 200 proteins not previously listed in ExoCarta reference set or in a comparable MS‐based SF‐EV proteomic study (Foers et al. 2020; Kugeratski et al. 2021) is overlaid onto the same HPA categories, as indicated by the numbers in circles. Insets highlight selected examples of this newly identified SF‐EV protein subset. (c) Functional‐group annotation of SF‐EV proteins within each HPA compartment, based on curated protein sets (HPA/KEGG/UniProt) as displayed. Selected representative proteins from indicated functional groups are highlighted in insets. Canonical EV‐associated proteins are displayed as a separate compartment‐independent functional group (green inset). (d) Overlap of the SF‐EV proteome with curated disease‐ and drug target‐associated protein sets (HPA/GeDiPNet), stratified by HPA compartment. Numbers in parentheses indicate the total size of each curated reference set, while numbers within the histograms display the number of overlapping SF‐EV proteins.

To further characterise the molecular composition of the arthritic SF‐EV proteome, we annotated all 3306 identified proteins using the Human Protein Atlas (HPA) reference set of 20,082 functionally annotated proteins (V 21.1 October 2024) (Digre and Lindskog 2023) and grouped them into three broad cellular compartment classes: proteins localised to the intracellular space, proteins associated to the cellular membrane system(s), and actively secreted proteins carrying a signal peptide. This classification provided a structured overview of proteins consistent with intravesicular cargo, EV membrane‐associated features, and extracellular or soluble proteins that may be present as luminal cargo or associated with the EV surface. Using this HPA‐based classification, 1966 proteins were assigned to the intracellular class (HPA‐I; 59.5%), 818 proteins to the membrane class (HPA‐M; 24.7%) and 514 proteins to the secreted class (HPA‐S; 15.6%), while eight proteins were not assigned in HPA (no HPA, 0.2%). The 200 proteins not previously described in MS‐based SF‐EV datasets were distributed across all three HPA classes, comprising 79 HPA‐I, 70 HPA‐M and 51 HPA‐S proteins, further indicating that the expanded proteome coverage was not restricted to a single protein compartment (Figure 5b, Table S2B).

Functional grouping of the HPA classes using curated protein sets from HPA, KEGG and UniProt highlighted the expected complexity of the SF‐EV proteome while providing a concise overview of the major protein families represented in the EV preparations (Figure 5c). Given that our refined workflow isolated EVs predominantly ranged from 30 to 120 nm, we expected enrichment of canonical small EV core proteins. Consistent with published data distinguishing small versus large EVs (Lischnig et al. 2022), the SF‐EV proteome contained a broad representation of intrinsic EV proteins linked to vesicle biogenesis, endosomal trafficking and secretion (Figure 5c, canonical EV proteins). This included ESCRT complex components, SNARE‐associated proteins, multiple Ras superfamily of small GTPases, and additional vesicle‐associated protein families such as annexins, flotillins, septins, tetraspanins, solute carrier transporters (SLCs), 14‐3‐3 family proteins (YWHAs) and RNA binding proteins (RBPs) implicated in (micro)RNA packaging/transfer reported in EV proteomes (O'Brien et al. 2020).

At the same time, the identified protein repertoire reflected the expected biological complexity of arthritic synovial fluid. The intracellular class highlighted multiple cytoskeleton proteins, consistent with EV release from activated and motile cells. A dominant fraction of metabolic enzymes was observed, supporting the concept that EVs mirror aspects of cellular physiology by carrying active enzymes and potentially associated metabolites. Additional prominent groups comprised chaperones, ribosomal/translation‐associated proteins and proteasome/ubiquitin‐related proteins, as well as selected kinases and transcriptional regulators several of them linked to inflammatory signalling in arthritic synovium (Figure 5c; HPA‐I). Given that synovial fluid contains substantial plasma ultrafiltrate and inflammatory exudate, especially in active arthritic conditions (e.g., swollen joints), the secreted class contained abundant plasma‐derived proteins, blood coagulation and fibrinolysis factors, complement components, acute‐phase proteins and immunoglobulin‐derived peptides. In addition, numerous ECM constituents and ECM‐modifying proteins were detected, consistent with the matrix‐rich and tissue‐remodelling environment of arthritic joints. Notably, a smaller set of soluble signalling proteins was also identified that are often challenging to capture in untargeted MS approaches, including inflammatory mediators (MIF, CCL18, TNFSF13), adipokines (ADIPOQ, RETN), growth factors (TGFB1, TGFB‐ligand GREM1) and WNT‐signalling proteins (WNT5A/B) (Figure 5c; HPA‐S).

The membrane‐associated protein class was particularly relevant for the broader aims of this study because surface‐associated proteins can support EV cell‐of‐origin attribution analysis and serve as candidates for future immunocapture strategies. As EV surface proteins represent key factors for functional interactions and immune‐related communication (Jahnke and Staufer 2024), the membrane class was enriched for receptors, receptor‐associated proteins, cell adhesion molecules and transporters. Among a set of 70 membrane‐associated proteins not previously reported in MS‐based SF‐EV datasets, we identified several receptors with relevance to immune regulation and inflammatory joint biology, including adhesion GPCRs (ADGRE2/3), cytokine/chemokine receptors (ILR3RA, CXCR1/2) and complement‐ and IgG superfamily receptors (C3AR1, TREM1, LILRA6/B5, LAIR1) (Figure 5c; HPA‐M).

Collectively, these observations illustrate that the SF‐EV proteome is enriched for proteins with established arthritis‐ and immune‐related relevance. An additional disease‐context was provided by intersecting the 3306‐protein dataset with curated disease‐ and drug target‐associated protein sets. The SF‐EV proteome yielded substantial overlap with proteins annotated for general arthritis, rheumatoid arthritis and immune diseases, alongside mappings to cancer‐associated proteins and FDA‐approved drug targets, thereby underlining the rationale that SF‐EVs carry a disease‐relevant molecular repertoire spanning inflammatory, immune and tissue remodelling biology (Figure 5d; Table S2C). Together, these results show that SEC‐UF‐enriched SF‐EVs provide broad EV‐associated proteome coverage while preserving molecular features consistent with the inflammatory, plasma‐derived and matrix‐rich environment of arthritic synovial fluid. This proteomic depth provided the basis for subsequent fraction‐resolved comparison of CD90‐immunocaptured and non‐captured SF‐EV populations.

### 3.5 Fraction‐Resolved Differential Proteomics Distinguishes CD90‐Immunocaptured Versus Non‐Captured SF‐EV Populations

Having established broad EV‐associated proteome coverage in SEC‐UF‐enriched SF‐EV preparations, we next evaluated whether CD90‐directed immunocapture resolves molecularly distinct EV populations from the heterogenous EV pool of patient synovial fluid. We performed MS‐based proteomic profiling of paired eluate (E) and flowthrough (FT) fractions generated from synovial fluid samples of 11 arthritis patients. The E fraction represents the CD90‐immunocaptured EV population, whereas the FT fraction contains the non‐captured CD90^−^/CD90^low^ SF‐EV pool. Principal component analysis (PCA) of the fraction‐resolved proteomic data showed separation of E and FT samples into distinct clusters (Figure S5a). This separation was less pronounced than in a parallel dataset of EVs derived from ex vivo‐cultured synovial fibroblasts, consistent with a more complex proteomic landscape of EVs derived from synovial fluid compared to cell culture CM.

We then interrogated the protein composition of the two fractions by differential expression analysis. In total, 616 proteins differed significantly between E and FT (adjusted *p* value < 0.05). Applying an additional effect‐size cutoff of |log_2_FC| ≥ 1.5 assigned 269 proteins as enriched in the CD90^+^ EV fraction and 296 proteins as enriched in the FT fraction, while 51 proteins did not meet the predefined effect‐size threshold despite statistical significance (Figure 6a; Figure S5b). Importantly, CD90 itself was enriched in the E fraction, confirming successful target‐based recovery at the proteomic level and supporting the Western blot‐based validation of CD90^+^ EV enrichment. Together, these results demonstrate that SEC‐UF‐coupled CD90 immunocapture enriches a target‐marker‐positive EV fraction and also resolves proteomically distinct EV populations from patient synovial fluid.

**FIGURE 6:**
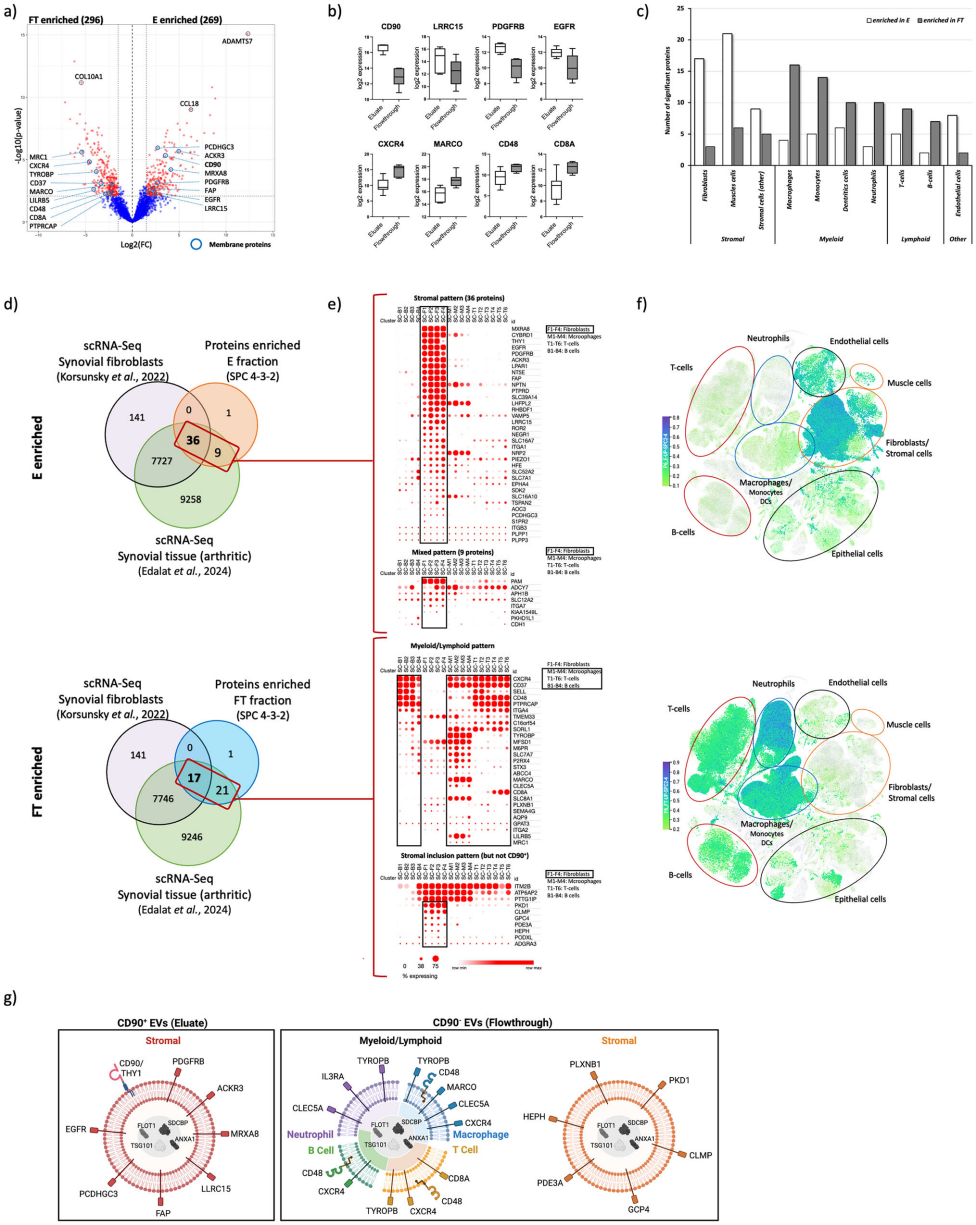
Surfaceome‐informed cell‐of‐origin profiling links eluate‐ and flowthrough‐enriched SF‐EV proteins to stromal‐ versus immune‐associated cell signatures. (a) Volcano plot of differential protein abundance between the CD90‐immunocaptured synovial fluid (SF) EV eluate fraction (E) and the non‐captured CD90^−^ flowthrough fraction (FT). Protein's meeting significance and effect‐size thresholds (FDR < 0.05; |log_2_FC| ≥ 1.5) are highlighted (red dots); selected membrane‐associated proteins are annotated as indicated. (b) Box plots showing log_2_‐normalised abundance values for representative proteins enriched in the E fraction (upper panel) or FT fraction (lower panel), as indicated. Box plots show median and interquartile range (IQR); whiskers indicate the 5th–95th percentiles. (c) Distribution of significantly enriched surface proteins across curated cell‐type signature (CTS) groups. Proteins were restricted to those with cell surface protein consensus (SPC) scores 4–3–2 and assigned to 10 CTS groups; counts are shown for E‐ versus FT‐enriched proteins, as indicated. (d) Venn diagrams comparing E‐ and FT‐enriched protein sets (SPC score 4–3–2) with published synovial fibroblast and synovial tissue single‐cell transcriptomic reference datasets (Korsunsky et al. 2022; Edalat et al. 2024), as cited in the main text). (e, f) Projection of E‐ and FT‐enriched proteins onto two independent single‐cell gene expression atlases: (e) Broad Institute Single Cell Portal (SCP; AMP Phase I RA synovial tissue dataset; Zhang et al., 2019) and (f) Tabula Sapiens. Dot plots/UMAP representations show the distribution of mapped proteins (as shown in Panel d) across annotated cell types as displayed in each atlas. In Panel e, dot size denotes the proportion of cells within a given cell type annotation expressing the corresponding gene, and dot colour density indicates the average expression level, as displayed. In Panel f, coloured circles in UMAP plots depict annotated cell type clusters. (g) Schematic summary of membrane proteins enriched in the CD90‐immunocaptured SF‐EV subpopulation (Eluate fraction) versus membrane proteins enriched in the heterogenous pool of non‐captured CD90^−^ SF‐EV populations (flowthrough fraction). Figure created with BioRender.com.

### 3.6 Surfaceome‐Informed Cell‐of‐Origin Profiling Resolves Stromal‐Associated Versus Broader Immune‐Associated EV Inputs in Eluate and Flowthrough Fractions

Differential proteomic analysis indicated that CD90‐directed immunocapture separates the heterogeneous SF‐EV pool into molecularly distinct E and FT fractions. To further contextualise these differences and identify likely cellular contributors to each EV fraction, we implemented a surfaceome‐informed cell‐of‐origin profiling strategy focused on membrane‐associated proteins with evidence for cell‐surface localisation. This approach prioritises proteins with potential EV surface accessibility, which are therefore particularly informative for EV attribution and future immunocapture target discovery. Initial inspection of established cell type‐associated markers among the differentially enriched proteins supported the enrichment of stromal‐associated EV features in the CD90^+^ EV fraction. In addition to CD90, the E fraction was co‐enriched for stromal‐associated surface proteins including FAP, PDGFRB and EGFR. In contrast, the FT fraction contained immune‐associated markers such as CXCR4, CD8A, CD48 and MARCO, consistent with broader myeloid and lymphoid EV inputs in the non‐captured SF‐EV pool (Figure 6b).

The representation of stromal‐ and immune‐associated markers in SF‐EVs was further supported by the analysis of annotated CD proteins. Of 384 curated CD markers, 150 were detected in the SF‐EV proteomic dataset, including seven markers significantly enriched in E and 10 enriched in the FT fraction. In analogy to synovial tissue scRNA‐Seq studies (Zhang, Jonsson, et al. 2023), these CD markers were allocated along a myeloid‐lymphoid‐stromal continuum (Table 1, CD markers). To extend this analysis beyond CD proteins, we curated a panel of 10 cell type signatures (CTS) from HPA and PangLaoDB gene sets, representing arthritis‐relevant cellular compartment (Table S4). These CTS included macrophage/monocyte, dendritic cell and granulocyte/neutrophil signatures; T‐cell, NK‐cell and B‐cell signatures; fibroblast, muscles cell and a group of additional stromal/joint‐resident signatures related to adipocytes, chondrocytes, osteoblasts and osteocytes. An endothelial signature was also included to account for vascular and perivascular contributions within arthritic synovial tissue.

**TABLE 1:** Selected CD markers (*n* = 150) detected in synovial fluid EVs from arthritis patients and grouped by cell‐type association.

Stromal		Stromal shared with myeloid/lymphoid		Myeloid		Myeloid/lymphoid shared		Lymphoid	
CD‐name	Gene name	CD‐name	Gene name	CD‐name	Gene name	CD‐name	Genename	CD‐name	Gene name
CD10	MME	CD36	FAT	CD14	CD14	CD37	TSPAN26	CD3E	CD3
CD34	CD34	CD39	ENTPD1	CD16	FCGR3A	CD43	SPN	CD4	CD4
CD90	THY1	CD54	ICAM1	CD32a	FCGR2A	CD44	HCELL	CD8A	CD8
CD99	MIC2	CD55	DAF	CD46	MCP	CD45	PTPRC	CD26	DPP4
CD140a	PDGFRA	CD57	BST1	CD64	FCGR1A	CD48	SLAMF2	CD38	ADPR1
CD140b	PDGFRA	CD91	LRP1	CD66a	CAECAM1	CD50	ICAM3	CD40	TNFRDF5
CD151	TSPAN24	CD93	C1QR1	CD66b	CAECAM18	CD53	TSPAN25	CD73	NT5E
CD167b	DDR2	CD95	FAS	CD68	LAMP4	CD62L	SELL	CD138	SDC1
CD201	PROCR	CD101	IGSF2	CD85c	LILRB5	CD84	SLAMF5	CD197b	IGLL1
CD276	B7H3	CD105	ENG	CD85d	LILRB2	CD97	ADGRE5	CD307e	FCRL5
CD280	MRC2	CD106	VCAM1	CD87	PLAUR	CD143	ACE		
		CD109	CPAMD1	CD88	C5AR1	CD156a	ADAM8		
		CD112	NECTIN2	CD89	FCAR	CD162	SELPLG		
		CD141	THBD	CD115	CSF1R	CD181	CXCR1		
		CD248	TEM1	CD156b	ADAM17	CD182	CXCR2		
		CD304	NRP1	CD163	M130	CD184	CXCR4		
		CD315	PTGFRN	CD169	SIFLEC1	CD191	CCR1		
		CD340	ERBB2	CD170	SIGLEC5	CD195	CCR5		
		CD362	SDC2	CD177	NB1	CD225	IFITM1		
				CD191	CCR1	CD300a	IGSF12		
				CD206	MRC1	CD305	LAIR1		
				CD232	PLXNC1				
				CD282	TLR2				
				CD312	ADGRE2				
				CD354	TREM1				

Because cell type‐associated proteins are most informative for EV attribution and future marker‐directed immunocapture strategies when they are exposed on the vesicle surface, we restricted the subsequent analysis to membrane‐associated proteins with evidence for cell‐surface‐localisation. For this purpose, we applied the cell surface protein consensus (SPC) score, which ranges from 0 to 4 and estimates the likelihood of cell‐surface localisation based on curated surfaceome datasets (Waas et al. 2020; Zhang, Ma, et al. 2023). Proteins with SPC scores 4, 3 or 2 were classified as ‘cell surface localised’, whereas SPC 1 indicated ‘predicted localisation’ and SPC 0 ‘no evidence’ for cell‐surface localisation. This filtering strategy identified 559 SF‐EV proteins with SPC scores 4–3–2, including 90 significantly enriched proteins across the E and FT fractions. Of these, 46 were enriched in E, 39 in FT and 5 were statistically significant but did not meet the predefined effect‐size threshold (Table S3).

Cross‐referencing the significantly enriched surface‐localised proteins with the curated CTS panel revealed a prominent stromal‐associated signature in E, with matches to fibroblasts, muscle cells and broader stromal/mesenchymal signatures. In contrast, myeloid and lymphoid CTS were preferentially represented in the FT fraction (Figure 6c). The endothelial CTS also showed representation in E, indicating that the CD90^+^ EV fraction may include contributions from endothelial‐associated or perivascular cell states, such as mural cells or pericytes, which can share stromal‐vascular marker features in synovial tissue (Wei et al. 2020).

To further evaluate whether the surface‐localised proteins enriched in E and FT align with synovial tissue cell populations, we compared these protein sets with published scRNA‐Seq datasets of synovial fibroblasts (Korsunsky et al. 2022) and inflammatory arthritic synovium (Edalat et al. 2024) (Figure 6d). Among the 46 SPC 4–3–2 proteins significantly enriched in E, 36 overlapped with the synovial fibroblast reference dataset, corresponding to 78% of the E‐enriched surface‐localised protein set. This overlap included CD90, ACKR3, EGFR, FAP, LRRC15, MXRA8, NRP2, PCDHGC3 and PDGFRB. The E‐enriched set also contained additional surface‐localised proteins, not typically highlighted as canonical stromal‐associated markers, that merit future evaluation as candidate markers for stromal‐associated EV subset characterisation, including EPHA4, LPAR1, MELTF, NPTN, NT5E, PTPRD and ROR2. By comparison, among the 39 SPC 4–3–2 proteins significantly enriched in FT, 17 overlapped with the synovial fibroblast reference dataset, corresponding to 44% of the FT‐enriched surface‐localised protein set. The remaining non‐overlapping FT‐enriched proteins included markers commonly linked to lymphoid and myeloid lineages, such as CD8A, PTPRCAP, CXCR4, MRC1, CD48, CD37, LILRB5, M6PR, MFSD1, MRP4/ABCC4, SLC7A7 and CLEC5A. Together, these patterns support the interpretation that CD90‐directed immunocapture enriches a stromal‐associated EV fraction, whereas the non‐captured CD90^−^/CD90^low^ EV pool in FT retains a broader immune‐associated EV signature together with residual stromal contributions. This interpretation is further supported by FT‐enriched proteins intersecting with the synovial fibroblast reference dataset, including, PDE3A, HEPH, ADGRA3 or GPC4, consistent with the presence of residual CD90^−^/CD90^low^ stromal‐associated EVs in the non‐captured EV pool. Endothelial‐associated proteins such as CLMP and PODXL were also detected in FT, indicating that this fraction remains compositionally heterogenous rather than representing a purely immune‐associated EV population.

Finally, we projected the corresponding genes for E‐ and FT‐enriched surface‐localised proteins onto synovial tissue and whole‐body single‐cell gene expression resources to further validate the cell‐contributor patterns observed in the CTS analysis. To represent different synovial cell states in inflammatory arthritis, we used the Broad Institute Single Cell Portal (SCP) implementation of the Accelerating Medicines Partnership (AMP) Phase I project data set for arthritic synovial tissue (data set 1 Rheumatoid Arthritis [Tarhan et al. 2023; Zhang et al. 2019]). To assess broader tissue‐level expression patterns, we additionally queried the Tabula Sapiens huma cell atlas (Tabula Sapiens et al. 2022). Projection of E‐enriched proteins onto the synovial tissue cell atlas revealed a predominantly fibroblast/stromal expression pattern, whereas FT‐enriched proteins displayed a mixed myeloid/lymphoid pattern with stromal inclusions (Figure 6e, Figure S6a,b). A similar separation of E‐ and FT‐enriched protein sets was observed in the Tabula Sapiens atlas (Figure 6f). This pattern was further supported by extracting quantified expression values for selected cell types from the CZ CELLxGENE portal and visualising them in a radar distribution blot using cell types that mirrored the curated CTS panel (Figure S6c).

Collectively, surfaceome‐informed marker analysis, curated CTS mapping and projection onto synovial and whole‐body single‐cell atlases converged on a consistent cell‐contributor pattern across the two fractions. The CD90^+^ EV fraction E was characterised by a prominent stromal‐associated surface signature with an additional endothelial/perivascular component, whereas the non‐captured CD90^−^/CD90^low^ EVs in FT retained broader myeloid and lymphoid immune‐associated contributions with residual stromal signals (Figure 6g). These results provide a marker‐based validation of the fraction‐resolved proteomic differences and support CD90‐targeted immunocapture as strategy to enrich a stromal‐associated EV subpopulation from heterogenous arthritic synovial fluid. Together, differential proteomic profiling and surfaceome‐informed cell‐of‐origin analysis provide a focused validation that supports the use of targeted immunocapture to molecularly dissect EV heterogeneity in complex biofluids and establish an analytical framework for future biomarker‐oriented studies of defined EV subpopulations in arthritis.

## 4 Discussion

Extracellular vesicles (EVs) are increasingly investigated as biofluid‐accessible carriers of disease‐associated molecular information and emerged as promising sources for biomarker development across diverse pathological conditions. In arthritis, this concept is particularly attractive because EVs encompass protein cargo and other bioactive components that may contribute to disease progression by modulating pathogenetically relevant processes (Schioppo et al. 2021; Boere et al. 2018; Foers et al. 2020; Mustonen and Nieminen 2021). This dual role, highlighting both biomarker potential and possible functional activity in disease pathogenesis, makes EVs attractive entry points for mechanistic insight and novel therapeutic concepts (Zhang et al. 2021). Because EVs and their cargo can reflect the physiological and pathological state of their cells and tissues of origin, they offer a window into joint pathobiology that is otherwise difficult to access in a minimally invasive manner. This is particularly relevant in the context of rheumatoid arthritis (RA), because the inflamed synovial tissue contains diverse functional subsets of synovial fibroblasts, macrophages and infiltrating immune cells, with distinct contributions to disease mechanisms and potentially also treatment response (Zhang, Jonsson, et al. 2023; Alivernini et al. 2020; Lewis et al. 2019). The ability to identify and resolve distinct EV populations in liquid biopsies from synovial fluid or plasma that reflect these disease‐relevant synovial cell states would therefore provide an attractive complement and less invasive alternative to tissue biopsies. Synovial fluid is in direct contact with the synovial tissue compartment and thus offers a minimally invasive specimen in which disease‐relevant EV populations may be sampled. In the present study, we developed a refined workflow that combines SEC‐UF enrichment with CD90‐targeted immunocapture to enrich and molecularly characterise a stromal‐associated EV subpopulations from arthritic synovial fluid. This strategy was not designed to replace tissue‐level profiling, but to provide a complementary approach for resolving selected EV subsets from a complex joint‐associated biofluid.

EV biomarker discovery in arthritis faces substantial technical obstacles, particularly when working with patient‐derived synovial fluid. A central challenge for SF‐EV profiling relies in the complexity of the source material. Synovial fluid contains a highly heterogenous mixture of EV populations originating from periarticular tissue‐resident, infiltrating and fluid‐associated cell sources (Schioppo et al. 2021; Zhang, Duan, et al. 2023). This complicates the attribution of bulk EV signature to specific disease‐driving cell states. In addition, patient‐to‐patient variability and dynamic disease activity may alter both EV composition and the physicochemical properties of synovial fluid (Ben‐Trad et al. 2022). These factors limit standardisation and complicate comparability between isolation methodologies. Moreover, synovial fluid is a protein‐rich lubricant with a distinct ECM composition and, during active arthritis, contains immune‐derived secretions and high‐abundance plasma proteins (Hui et al. 2012). Especially, plasma proteins and ECM fragments arising from inflammatory exudates and dysregulated tissue remodelling can constitute a substantial fraction of total protein measured in SF‐EV preparations (Toth et al. 2021; Boere et al. 2016), which can reduce the performance of affinity‐based isolation strategies and may increase the risk that low‐abundance EV‐associated proteins of interest are obscured. This is particularly relevant for downstream MS‐based proteomics, where wide protein concentration ranges can limit the sensitivity and the detection of less abundant proteins (Anderson et al. 2020). Several pre‐processing and purifications strategies have been proposed to mitigate these constraints, including hyaluronidase treatment of cell‐free synovial fluid to reduce viscosity and facilitate EV isolation (Boere et al. 2016), proteinase K treatment to remove ‘contaminating’ non‐nascent EV proteins from the EV surface (Foers et al. 2018), and selective affinity‐based depletion of abundant components such as IgG or proteoglycans (Singh et al. 2020). While such strategies can reduce co‐isolated or surface‐associated background proteins, they may also reduce EV yield or perturb biologically relevant surface proteins and EV‐associated corona components described for biofluid‐derived EVs (Toth et al. 2021; Buzas 2022). This is important because the EV surface represents a key interface for functional interactions, including immune modulation via exposed receptors and ligands that support paracrine and autocrine signalling (Jahnke and Staufer 2024). In immune cell‐derived EVs, corona‐associated proteins may contribute to EV uptake and immune cell engagement, and their loss has been associated with reduced functional interaction (Wolf et al. 2022; Liam‐Or et al. 2024; Dietz et al. 2023). EV surfaces can act as antigen‐presenting and immune‐complex‐binding hubs by displaying MHC class I/II, Fcgamma receptors and coupled immunoglobulins. In arthritic settings, EV surfaces may also carry or adsorb inflammatory mediators, DAMPs, active matrix‐modulating proteins or posttranslationally modified antigens relevant to autoimmunity such as citrullinated vimentin (Hallal et al. 2022; Kalluri 2024). These considerations support the use of EV enrichment workflows that reduce matrix complexity, while still be sufficiently gentle to preserve relevant EV surface proteins and corona‐associated features for downstream subpopulation analysis.

To address these challenges and preserve corona‐associated proteins that reflect the arthritic milieu while still enabling the characterisation of EV cargo and surface markers, we combined SEC‐UF EV enrichment with subsequent immunocapture of a selected SF‐EV subpopulation. The SEC‐UF pre‐enrichment reduces the abundance of loosely associated soluble background while generating an input material compatible with magnetic bead‐based Immunocapture. In addition, the workflow incorporated gravitational washing, during SEC and magnetic bead separation, that support a gentle removal of background material without disrupting the biologically informative vesicle surface and associated features. Compared with conventional bulk EV isolation, SEC‐UF‐coupled immunocapture integrates EV enrichment with targeted analysis of a defined EV subset, using a surface‐accessible target marker. Immunocapture is well established in cell separation using antibody‐coupled magnetic‐beads and has been adapted for EV isolation using diverse bead formats and technologies. Previous strategies have targeted canonical EV markers (CD63, CD9, CD81) (Karimi et al. 2022; Mathieu et al. 2021) or abundant broadly exposed molecules on the EV surface, such as phophatidylserine (Nakai et al. 2016), or disease‐ and cell‐type‐associated proteins such as KIT (mast cell EVs) (Pfeiffer et al. 2022), NRXN3 (CSF‐derived EVs) (Ter‐Ovanesyan et al. 2024), L1CAM (plasma‐ or neuron‐derived EVs) (Gilboa et al. 2024, Dunlop et al. 2023) and EPCAM1 (epithelial cancer EVs) (Kalra et al. 2013). In the present study, we selected CD90/THY1 as a proof‐of‐concept stromal‐associated marker because it is expressed by synovial fibroblasts subsets implicated in arthritic synovial biology (Zhang et al. 2019; Mizoguchi et al. 2018; Mimpen et al. 2023), and was detectable in arthritic SF‐EV preparations during the initial marker pre‐screening. Using anti‐CD90 antibodies coupled to prototype nanosised magnetic beads enabled the capture of CD90^+^ stromal‐associated EV subset from patient synovial fluid.

A relevant technical finding of this study was that the refined matrix‐adapted SEC‐UF pre‐enrichment improved the robustness of CD90‐directed EV recovery from patient synovial fluid. Direct immunocapture from pre‐treated synovial fluid showed variable outcomes in the samples tested, with increased albumin co‐enrichment and incomplete removal of non‐target markers. These findings are consistent with the complex composition of synovial fluid, where abundant soluble proteins, ECM fragments, protein aggregates and biofluid‐associated components may interfere with antibody accessibility or capture specificity. These observations emphasise that workflows validated in cell culture‐derived EV systems cannot be transferred unchanged to complex clinical biofluids. For synovial fluid, preparatory steps reducing the matrix complexity appears important, especially when target EV populations are expected to be low abundance or embedded within a highly heterogeneous EV and protein background.

Beyond validating CD90^+^ EV enrichment, MS‐based proteomics provided a quality and protein coverage assessment of the SEC‐UF‐enriched SF‐EV preparations, demonstrating that the workflow yields sufficient EV proteome depth for downstream comparison of distinct EV fractions. In addition, the proteomic assessment also situated the identified 3306 proteins within the biological context of the arthritic joint environment. The SF‐EV proteome from arthritis patients included a broad repertoire of diverse functional protein families. Next to canonical EV‐associated proteins, high‐abundance plasma‐derived proteins, complement factors, coagulation proteins, acute‐phase proteins, immunoglobulin‐related, ECM‐associated and matrix‐remodelling components were detected. Rather than treating these signals as contaminants, they should be interpreted in the context of the synovial fluid environment, where vesicular cargo, surface‐associated proteins, and co‐enriched biofluid‐derived components coexist. This is particularly relevant for inflammatory joint conditions, in which vascular leakage of plasma ultrafiltrate and inflammatory exudate, infiltrating immune cells and active matrix‐turnover shape the molecular composition of the synovial fluid and EV preparations. The identification of additional 200 proteins not previously reported in MS‐based SF‐EV datasets further indicated that SEC‐UF enrichment coupled to downstream proteomics can extend SF‐EV proteome coverage. Notably, many of these proteins are relevant to inflamed synovial environment and chronic degenerative processes in arthritis, including inflammation‐associated factors linked to DAMP signalling und inflammasome activation such as ALOX5, NLRC4 and CASP1/4 (Pinto et al. 2025; Delgado‐Arevalo et al. 2022); ECM‐turnover‐related metalloproteinases including ADAMDEC1 and ADAMTS7 (Li et al. 2019); the TRAIL decoy receptor 1 (TNFRSF10C), which functions as antagonistic receptor protecting cells from TRAIL‐induced apoptosis (Bisgin et al. 2010); and the anti‐inflammatory receptor LAIR1 (Zhang et al. 2018), which has been reported to be shed by activated synovial fibroblasts. These examples support the concept that the SF‐EV proteome contains potential biomarker candidates relevant to inflammation, immune regulation, matrix remodelling and chronic joint pathology. Validation in future clinically stratified cohorts will be needed to assess the relevance of those biomarker candidates in terms of disease activity, progression and therapeutic response.

Fraction‐resolved proteomics and surfaceome‐informed cell‐of‐origin analysis provided an additional, workflow‐relevant layer of interpretation. The differential analysis of proteins enriched in the eluate (E) and flowthrough (FT) fractions indicated that the CD90‐targeted immunocapture enriches a molecularly distinct, stromal‐associated EV subpopulation from synovial fluid. The CD90^+^ EV fraction showed enrichment of a stromal‐associated signature, aligning with synovial fibroblast reference datasets, whereas the non‐captured CD90^−^/CD90^low^ FT retained broader immune‐associated features together with residual stromal, vascular and additional joint‐related signals. This separation pattern is consistent with the design of the targeted approach and heterogeneity of synovial fibroblast cell states in arthritic synovium, describing functionally different CD90^+^ and CD90^−^ fibroblast subsets. Therefore, CD90‐directed immunocapture is not expected to recover all stromal EVs or to generate a pure non‐stromal FT fraction.

Surfaceome‐informed analysis also highlighted a broader conceptual advantage of surface marker‐directed subpopulation profiling. By prioritising surface‐accessible membrane proteins and aligning them with cell‐type‐associated expression patterns, this analysis can support future selection of alternative or complementary immunocapture targets beyond CD90 or PDPN. Accordingly, the present work establishes a methodological bridge between tissue‐resolved synovial biology and targeted biofluid EV analysis. Thus, the synovial cell states defined in the tissue atlases can inform candidate EV surface markers, while EV‐enriched preparations can be used to examine whether such markers are recoverable in patient‐derived biofluid. This does not establish direct equivalence between tissue cell states and EV subpopulations, but provides a practical roadmap for future studies aimed at refining liquid‐biopsy strategies in arthritis.

The present study has several limitations. First, although the workflow was applied to patient‐derived synovial fluid, the cohort size was limited and included different arthritis samples with expected biological heterogeneity. The study was therefore not designed to establish disease‐specific biomarkers, to distinguish different arthritis subgroups, or to relate EV features to clinical disease activity or treatment response. Such comparisons will require future studies with dedicated analyses in larger and clinically stratified cohorts. Secondly, healthy synovial fluid controls were not included. Availability of and access to healthy synovial fluid is limited by practical and ethical considerations and aspiration of non‐diseased joints is not routinely indicated. Third, we did not directly compare the SF‐EV proteome with matched unfractionated synovial fluid proteomics. Thus, the present data support targeted SF‐EV profiling as a complementary approach to conventional synovial fluid and tissue‐based analyses. Finally, while the presented workflow aims to preserve EV surface and corona‐associated proteins, the relative contributions of true vesicular cargo and surface‐bound proteins versus co‐isolated biofluid‐associated material cannot be fully resolved by proteomics alone and will require additional analytical approaches, such as targeted proteomics, imaging‐based validation, and functional assays.

In summary, this study establishes SEC‐UF‐coupled CD90‐directed immunocapture as a refined workflow strategy for enriching stromal‐associated EV subpopulations from complex arthritic synovial fluid. The approach combines matrix‐adapted EV pre‐enrichment with marker‐directed subpopulation recovery and provides sufficient proteomic depth to assess both bulk SF‐EV preparations and fraction‐resolved differences between captured and non‐captured EV subsets. Differential proteomics and surfaceome‐informed cell‐of‐origin analysis support the technical validity and biological context of the approach. Collectively, the presented findings provide a methodological and analytical approach for resolving EV heterogeneity in arthritic biofluids. Future studies in larger and clinically stratified cohorts will be required to determine whether CD90^+^ EVs or other defined EV subpopulations can support liquid biomarker discovery in arthritis.

## Author Contributions

A.N.T. and D.K. conceived the study, designed the experiments, wrote and approved the manuscript. S.K. and A.N.T. performed experiments and data analysis required for this study. S.K. wrote and approved the manuscript. D.K. provided funding and interpreted clinical data. E.H., S.H.‐M. and S.G. performed experiments, read and approved the manuscript. K.B. performed LC‐MS/MS measurement, read and approved the manuscript. F.G. and D.B. performed bioinformatic data analysis, read and approved the manuscript. S.W. and U.H. provided prototype magnetic beads and material, read and approved the manuscript. Y.P.A. and C.E. provided patient synovial tissue biopsies, read and approved the manuscript.

## Funding

This work is supported by the Swiss National Science Foundation (SNSF: 320030_197677 to D.K.).

## Ethics Statement

The study was approved by the ethics committee of Northwest and Central Switzerland (EKNZ No. 2019‐01693).

## Conflicts of Interest

S.W. and U.H. are employees of Miltenyi Biotec. All authors declare no conflict of interest.

## Supporting information




**Supplementary material**: jev270353‐sup‐0001‐SuppMat.docx

**Supplementary Table**: jev270353‐sup‐0001‐Tables.xlsx

**Supplementary Figure**: jev270353‐sup‐0001‐FigureS1.tiff

**Supplementary Figure**: jev270353‐sup‐0004‐FigureS2.tiff

**Supplementary Figure**: jev270353‐sup‐0005‐FigureS3.tiff

**Supplementary Figure**: jev270353‐sup‐0005‐FigureS4.tiff

**Supplementary Figure**: jev270353‐sup‐0006‐FigureS5.tiff

**Supplementary Figure**: jev270353‐sup‐0006‐FigureS6.tiff

## Data Availability

All data relevant to the study are available upon reasonable request and are uploaded to online data repositories. Raw MS data are available on ProteomeXchange with the identifier PXD063496. Processed MS data and analysis scripts are available from https://doi.org/10.5281/zenodo.15227820.
