# Supplementary material for: Enrichment and Proteomic Profiling of Stromal‐Associated Extracellular Vesicle Subpopulations From Arthritic Synovial Fluid by Size‐Exclusion Chromatography Coupled to CD90‐Directed Immunocapture
Source: J Extracell Vesicles. 2026 Sep 30;15(10):e70353. doi: 10.1002/jev2.70353 (PMC13626482; doi:10.1002/jev2.70353)
Supplement: Supplementary file 1 [file JEV2-15-e70353-s002.docx]

**Supplementary material – Table legends**

**Table S1: Clinical characteristic for synovial fluid and synovial tissue fibroblast samples.** Columns denote the following information for each sample: clinical diagnosis, treatment at time of sample, seropositivity status by positive or negative measurement of rheumatoid factor (RF) and anti-cyclic citrullinated peptide antibody (Anti-CCP) in patient blood, inflammation status by measurement of c-reactive protein (CRP) levels in patient blood, white cell count in synovial fluid, EULAR disease activity score 28 (DAS28) for classifying the severity of rheumatoid arthritis, sex and age (in years).

**Table S2A: List of LC-MS/MS-identified extracellular vesicle proteins isolated from synovial fluid (N=11) or conditioned medium of cultured synovial fibroblasts (FLS, N=10) samples.** All samples were measured in the same mass spectrometry run (total size= 3879). The presence of each protein in the corresponding synovial fluid and synovial fibroblast eluate (E) or flowthrough) (FT) sample group was assessed by a fraction missing value (FMiss). Proteins present in all samples of a sample group have a FMiss = 0, while proteins completely missing in a sample group have a FMiss = 1. Total size of synovial fluid sample proteins= 3306. Names for proteins (related gene name) derived from the bioinformatic analysis which are not assigned in Human protein atlas (HPA) database are change to the corresponding HPA identifier, or, if missing in HPA, are denoted as not assigned (na).

**Table S2B: Protein lists from reference datasets used for the overlap with SF-EV proteome in Figure 5a-b**. Table includes the protein lists derived from ExoCarta (Version 6, MS-measured small EV proteins), Human Protein Atlas (HPA, MS-measured high abundance plasma proteins), Foers *et al.* 2020 (MS-measured SF-EV proteins), and the list of 200 unique proteins identified in the current SF-EV proteome study not previously listed by the three reference datasets (stratified by HPA compartment).

**Table S2C: Protein lists used for disease overlay of the SF-EV proteome with curated disease- and drug target-associated protein sets in Figure 5d.** Table includes the protein lists derived from Human Protein Atlas (HPA) and GeDiPNet, separated in overlaid (stratified by HPA compartment) and not mapped SF-EV proteins from the current study.

**Table S3: List of 3306 differentially expressed proteins from LC-MS/MS-measured SEC-UF-enriched SF-EVs of arthritis patients comparing eluate (E) versus flowthrough (FT) fraction samples following CD90-directed immunocapture (N=11).** Data in columns are standard outputs from limma package including the corresponding Ensemble GeneID, Symbol and GeneName of the analyzed protein, the surface prediction consensus (SPC) score, the fold change expression (log2FC), the nominal p value (P.Value) and the adjusted p value (adj.P.Val). Names for proteins (related gene name) derived from limma output which are not assigned in Human protein atlas (HPA) database are change to the corresponding HPA identifier, or if missing in HPA are denoted as not assigned (na).

**Table S4: Curated gene list of 10 cell type signatures (CTS).** Curated gene lists representing 10 synovium-associated cell type signatures (CTS). Number in parenthesis represents the size of each individual gene set.
